# Entropic pressure controls the oligomerization of the *Vibrio cholerae* ParD2 antitoxin

**DOI:** 10.1107/S2059798321004873

**Published:** 2021-06-18

**Authors:** Gabriela Garcia-Rodriguez, Yana Girardin, Alexander N. Volkov, Ranjan Kumar Singh, Gopinath Muruganandam, Jeroen Van Dyck, Frank Sobott, Wim Versées, Daniel Charlier, Remy Loris

**Affiliations:** a Structural Biology Brussels, Vrije Universiteit Brussel, Pleinlaan 2, 1050 Brussels, Belgium; b VIB–VUB Center for Structural Biology, Vlaams Instituut voor Biotechnologie, Pleinlaan 2, 1050 Brussels, Belgium; c Jean Jeener NMR Center, Vrije Universiteit Brussel, Pleinlaan 2, 1050 Brussels, Belgium; d Department of Chemistry, Universiteit Antwerpen, Groenenborgerlaan 171, 2020 Antwerp, Belgium; e Astbury Centre for Structural Molecular Biology, University of Leeds, Leeds LS2 9JT, United Kingdom; f Research Group of Microbiology, Vrije Universiteit Brussel, Pleinlaan 2, 1050 Brussels, Belgium

**Keywords:** toxin–antitoxin module, ParD2, *Vibrio cholerae*, intrinsically disordered proteins, transcription regulation, oligomer interface, protein–DNA interactions, protein oligomers, quaternary structure

## Abstract

ParD2 is the antitoxin component of the *parDE2* toxin–antitoxin module from *Vibrio cholerae* and consists of an ordered DNA-binding domain followed by an intrinsically disordered ParE-neutralizing domain. In the absence of the C-terminal intrinsically disordered protein (IDP) domain, *V. cholerae* ParD2 (*Vc*ParD2) crystallizes as a doughnut-shaped hexadecamer formed by the association of eight dimers. This assembly is stabilized via hydrogen bonds and salt bridges rather than by hydrophobic contacts. In solution, oligomerization of the full-length protein is restricted to a stable, open decamer or dodecamer, which is likely to be a consequence of entropic pressure from the IDP tails. The relative positioning of successive *Vc*ParD2 dimers mimics the arrangement of *Streptococcus agalactiae* CopG dimers on their operator and allows an extended operator to wrap around the *Vc*ParD2 oligomer.

## Abstract 2

ParD2 forms an open oligomer in solution, the size of which is limited by the presence of an intrinsically disordered tail. In the absence of this tail, ParD2 forms a circular hexadecamer.

## 1. Introduction   

Like all organisms, bacteria have to deal with various kinds of stress that threaten their survival. These include nutrient starvation, chemical stress arising from compounds, including antibiotic or redox stress, and physical stress such as heat or salt. To deal with this, they have developed different strategies, including the generation of persister cells, a specific stochastically induced dormant metabolic state that allows a sub­population to survive antibiotics to which they are not resistant. Among the potential players in the bacterial stress-response network are sets of small two-gene operons encoding a toxic protein and a corresponding neutralizing protein or RNA, known as toxin–antitoxin (TA) modules. They are often abundant in free-living bacteria and opportunistic pathogens (Pandey & Gerdes, 2005 ▸). For example, the well known *Mycobacterium tuberculosis* contains at least 88 such modules, while the closely related *M. smegmatis* only contains five (Shao *et al.*, 2011 ▸).

The physiological role of toxin–antitoxin modules has been greatly debated. One of their potential functions is the stabilization of mobile genetic elements (Gerdes *et al.*, 1986 ▸; Szekeres *et al.*, 2007 ▸). After initially having been observed as stabilizing elements on low-copy-number plasmids, TA modules were also found on chromosomal regions that do not encode essential genes, including integrative and conjugative elements, the superintegrons of *Vibrio cholerae* and *V. vulnificus*, chromosome II, cryptic prophages and genomic and pathogenicity islands (Díaz-Orejas *et al.*, 2017 ▸; Yao *et al.*, 2018 ▸).

The most popular proposed function for TA modules is stress response (Gerdes, 2000 ▸; Gerdes *et al.*, 2005 ▸; Hõrak & Tamman, 2017 ▸). Indeed, the activity of TA toxins is upregulated during episodes of stress. This was initially proposed to be a consequence of the protease-dependent degradation of antitoxins, a mechanism that has recently been challenged (LeRoux *et al.*, 2020 ▸; Song & Wood, 2020*a* ▸). Furthermore, it remains unclear whether stress-related TA activation is an integral part of the stress-response network or whether it is rather a side effect of the SOS response, which is the general bacterial response to DNA damage (Little & Mount, 1982 ▸). Equally, the supposed role of TA modules in the onset of persistence remains unclear (Ronneau & Helaine, 2019 ▸).

Another function that has been attributed to TA modules is protection against bacteriophages via abortive infection (Fineran, 2019 ▸; Lopatina *et al.*, 2020 ▸; Song & Wood, 2020*b* ▸). Last but not least, it should be considered that TA modules might be mere selfish genetic elements that have adapted and been conserved in a number of cases because they provide some additional beneficial properties such as the functions described above.

Toxin–antitoxin modules have been divided into eight classes based on the nature of the antitoxin (protein or RNA) and the mechanism by which it counteracts the toxin (Page & Peti, 2016 ▸; Song & Wood, 2020*a* ▸). The most common and best-studied class of toxin–antitoxin modules are the type II modules, in which the antitoxin encodes a protein that counteracts the toxin via the formation of a tight noncovalent complex. Type II modules can be further classified into an increasing number of nonrelated families. Among these, the *parDE* family of TA modules, although one of the first families to be discovered, has been relatively understudied (Roberts & Helinski, 1992 ▸). For two members, *parDE* from *Escherichia coli* plasmid RK2 and *parDE2* from *V. cholerae*, the target of the ParE toxin was identified as gyrase (Jiang *et al.*, 2002 ▸; Yuan *et al.*, 2010 ▸). Like the more intensively studied CcdB, ParE poisons gyrase by stabilizing the so-called cleavable complex between gyrase and DNA. This leads to double-strand breaks, activation of the SOS response and ultimately, if the ParE toxin is not counteracted by the antitoxin ParD, cell death.

Currently, the Protein Data Bank (PDB) contains an NMR structure of ParD from plasmid RK2, an NMR structure of *E. coli* PaaA2, which is a truncated ParD protein lacking a DNA-binding domain, and crystal structures of ParD–ParE complexes from *Caulobacter crescentus* and *Mesorhizobium opportunistum* and of the PaaA2–ParE2 complex from the *parDE*-like *paaR2*–*paaA2*–*parE2* module of *E. coli* O157:H7 (Oberer *et al.*, 2007 ▸; Sterckx *et al.*, 2014 ▸, 2016 ▸; Dalton & Crosson, 2010 ▸; Aakre *et al.*, 2015 ▸). The target has not been identified for either of the ParE proteins, and evidence has been presented that *E. coli* ParE2 does not interact with the DNA gyrase A subunit (Sterckx *et al.*, 2016 ▸). Plasmid RK2, *M. opportunistum* and *C. crescentus* ParD fold into an N-terminal ribbon–helix–helix DNA-binding domain that is followed by a domain which is unfolded in solution in plasmid RK2 ParD but folds into a helix–helix–strand conformation that wraps around the ParE toxin in *C. crescentus* and *M. opportunistum* ParD. *E. coli* PaaA2 lacks the N-terminal DNA-binding domain and is mostly disordered in solution, but adopts the same conformation as the C-terminal domain of *C. crescentus* or *M. opportunistum* ParD when bound to its cognate ParE2.

The genome of *V. cholerae* contains three *parDE* modules, all of which are located in the superintegron on chromosome II (Yuan *et al.*, 2011 ▸). The *parDE1* and *parDE3* modules have identical open reading frames and regulatory sequences, while the ParD and ParE proteins encoded by the *parDE2* module share 14% and 22% sequence identity, respectively, with their *parDE1*/*parDE3* counterparts. ParE2 has been shown to inhibit gyrase *in vitro* and to bind to an epitope on the gyrase A subunit that differs from the one targeted by F-plasmid CcdB (Yuan *et al.*, 2010 ▸). *In vivo*, both ParE1/ParE3 and ParE2 inhibit cell division, activate the SOS response and contribute to the degradation of chromosome I upon the loss of chromosome II.

Transcriptional regulation of TA modules is often complex and involves ratio-dependent interplay between the toxin and antitoxin (Garcia-Pino *et al.*, 2010 ▸; Yamaguchi & Inouye, 2011 ▸; Jurėnas *et al.*, 2019 ▸; Page & Peti, 2016 ▸; Vandervelde *et al.*, 2017 ▸; Xue *et al.*, 2020 ▸). Intrinsically disordered segments on the antitoxin or toxin often play a major role in these mechanisms (De Jonge *et al.*, 2009 ▸; Garcia-Pino *et al.*, 2016 ▸; Loris & Garcia-Pino, 2014 ▸; Talavera *et al.*, 2019 ▸; De Bruyn *et al.*, 2021 ▸). Little information has been obtained, however, on the regulation of *parDE* modules. Early work on plasmid RK2 *parDE* suggested that ParD alone can act as a repressor of the operon (Roberts *et al.*, 1993 ▸), but it is not known whether the ParE toxin modulates this action. Here, we describe the crystal structure of *V. cholerae* ParD2 (*Vc*ParD2) and show that inter-dimer contacts in the crystal mimic the functional arrangement of the structurally related *Streptococcus agalactiae* CopG bound to its DNA target (PDB entry 1b01; Gomis-Rüth *et al.*, 1998 ▸). While a doughnut-shaped hexadecamer is observed in the crystal, in solution smaller decamers and dodecamers are present that form a partial doughnut with otherwise similar inter-subunit contacts. The partial disruption of the full hexadecameric ring may possibly be attributed to steric pressure from the intrinsically disordered C-terminal tails that are absent in the crystallized entity.

## 2. Materials and methods   

### 2.1. Expression and purification   

The coding region of the *V. cholerae parDE2* operon was cloned into pET-28a, placing a T7 promotor upstream of the *parD2* gene and adding a His tag to the C-terminus of *Vc*ParE2. *E. coli* BL21 (DE3) cells were transformed with this vector and grown at 37°C in lysogeny broth supplemented with 50 µg ml^−1^ kanamycin. Expression was induced at an OD_600_ of 0.6 by adding 0.5 m*M* isopropyl β-d-1-thiogalactopyranoside. The cultures were incubated overnight at 20°C, after which the cells were harvested by centrifugation at 5000 rev min^−1^ and 4°C for 15 min.

The cells were resuspended in 20 m*M* Tris pH 8.0, 500 m*M* NaCl, 2 m*M* β-mercaptoethanol and lysed with a cell cracker after adding 50 µg ml^−1^ DNase and 2 m*M* MgCl_2_. After centrifugation at 19 500 rev min^−1^ and 4°C for 35 min, the supernatant was filtered using a 0.45 µm filter and then loaded onto a 5 ml HisTrap HP Nickel Sepharose column (GE Healthcare) equilibrated with the same buffer. The column was washed with 20 m*M* Tris pH 8.0, 500 m*M* NaCl, 2 m*M* β-mercaptoethanol, and the *Vc*ParD2–*Vc*ParE2 complex was subsequently eluted using a step gradient of imidazole (0, 10, 25, 50, 250 and 500 m*M*) in the same buffer. The resulting samples were loaded onto a Superdex 200 16/90 size-exclusion chromatography (SEC) column (GE Healthcare) pre-equilibrated with 20 m*M* Tris pH 8.0, 500 m*M* NaCl, 2 m*M* β-mercaptoethanol to obtain pure *Vc*ParD2–*Vc*ParE2 complex.

The *Vc*ParD2–*Vc*ParE2 complex was reloaded onto a Ni–NTA column followed by washing with 20 m*M* Tris pH 8.0, 0.5 *M* NaCl, 10% ethylene glycol. *Vc*ParD2 was eluted by applying a step gradient of guanidinium hydrochloride (GndHCl; 0, 2.5 and 5 *M*) in 20 m*M* Tris pH 8.0, 500 m*M* NaCl, 2 m*M* β-mercaptoethanol. The resulting *Vc*ParD2 fractions were pooled and dialyzed against 20 m*M* Tris pH 8.0, 150 m*M* NaCl prior to final SEC on a Superdex 200 16/60 column (GE Healthcare).

The Ni–NTA column was subsequently washed with (i) 20 m*M* Tris pH 8.0, 25 m*M* NaCl, 5% glycerol, (ii) 20 m*M* Tris pH 8.0, 150 m*M* NaCl, 5% glycerol and (iii) 20 m*M* Tris pH 8.0, 250 m*M* NaCl, 2 m*M* β-mercaptoethanol to refold *Vc*ParE2 on the column. *Vc*ParE2 was eluted using a step gradient of imidazole (25, 50, 250 and 500 m*M*) and was further purified on a Superdex 75 16/60 column. The purity of all samples was checked by SDS–PAGE and nano-electrospray ionization–time of flight (nESI-TOF) mass spectrometry. Macromolecule-production information is summarized in Table 1 ▸.

### 2.2. Electrophoretic mobility shift assay   

A 350 bp DNA fragment comprising the first 67 bp of the *parD2* ORF and extending further upstream into the control region of the *parDE2* operon from *V. cholerae* biovar El Tor strain N16961 (NCBI NC_002506.1) was synthesized by PCR-based gene assembly (Stemmer *et al.*, 1995 ▸) after optimization of the oligonucleotides using the *DNAWorks* platform (Hoover & Lubkowski, 2002 ▸). The assembled 350 bp fragment was used as the template for PCR amplification of a 151 bp fragment comprising the putative operator region upstream of the *parD2* ORF with the oligonucleotides forward (Fw) 5′-TGAGGCGTTTGTTATGCGC and reverse (Rv) 5′-TTTGTATTTGGCTTGTAATAAAGCCAT as primers, of which one was 5′-^32^P single-end labelled with (γ^32^P)-ATP (Perkin Elmer, 3000 Ci mmol^−1^) and T4 polynucleotide kinase (Thermo Fisher) as described by Nguyen Le Minh *et al.* (2018 ▸). Labelled PCR fragments were purified by gel electrophoresis on 6% polyacrylamide. For electrophoretic mobility shift assays (EMSAs), increasing concentrations of ParD2 were mixed with labelled DNA (10 000–15 000 cpm) in buffer consisting of 20 m*M* Tris pH 8, 150 m*M* NaCl, 1 m*M* TCEP in a total volume of 20 µl and incubated at 20°C for 30 min. After incubation, 3 µl loading buffer (25% Ficoll, 0.1% xylene­xyanol, 0.1% bromophenol) was added to each sample. Separation was performed on 6% polyacrylamide gels run in TBE buffer at 130 V for approximately 3 h.

### 2.3. Limited proteolysis   

*Vc*ParD2 at 20 µ*M* was incubated with different molar ratios of trypsin, proteinase K and subtilisin (1:10, 1:100, 1:1000 and 1:10 000) in 10 m*M* Tris–HCl pH 8.0, 5 m*M* CaCl_2_. The mixtures were gently vortexed and incubated at 25°C. Samples were taken at different time points (1 min, 15 min, 1 h and 2 h) and the reaction was stopped by adding quenching solution (0.1 m*M* leupeptin, 1 m*M* AEBSF, 1 m*M* CaCl_2_ and a cOmplete ULTRA protease-inhibitor tablet) followed by 15 min incubation on ice. The samples for SDS–PAGE analysis were prepared at 1:4 dilution with colourless 4× SDS–PAGE loading dye (200 m*M* Tris pH 6.8, 277 m*M* SDS, 40 m*M* glycerol, 50 m*M* EDTA), boiled for 5 min at 95°C and loaded onto a 4–20% Mini-PROTEAN TGX Precast gel (Bio-Rad).

### 2.4. Crystallization, data collection and structure determination   

The protein was concentrated to 20 mg ml^−1^ in 20 m*M* Tris pH 8.0, 150 m*M* NaCl. For screening crystallization conditions, 0.1 µl protein solution was mixed with 0.1 µl reservoir solution in a sitting drop and equilibrated against 100 µl reservoir solution using a Mosquito HTS robot (SPT Labtech). Crystals were observed after approximately three months in 0.2 *M* lithium sulfate, 0.1 *M* MES pH 6.0, 20%(*w*/*v*) PEG 4000. The crystals were transferred to precipitant solution supplemented with 25% PEG 400 for cryoprotection and were immediately flash-cooled in liquid nitrogen. Crystallization information is summarized in Table 2 ▸. Data were collected on the PROXIMA-2A beamline at the SOLEIL synchrotron facility, Gif-sur-Yvette, Paris, France. All data were indexed, integrated and scaled with *XDS* (Kabsch, 2010 ▸) via the *XDSME* interface (Legrand, 2017 ▸). The solvent content was analyzed using the *CCP*4 program *MATTHEWS_COEF* (Kantardjieff & Rupp, 2003 ▸). Data-collection and processing statistics are summarized in Table 3 ▸.

The structure of *Vc*ParD2 was determined by molecular replacement with *Phaser-MR* (McCoy, 2007 ▸) using the dimer of the N-terminal domain (residues 1–50) of *C. crescentus* ParD2 (*Cc*ParD2; PDB entry 3kxe; 63% sequence identity; Dalton & Crosson, 2010 ▸). Four copies of the N-terminal domain dimer were placed in the asymmetric unit. Several cycles of refinement in *Phenix* (Liebschner *et al.*, 2019 ▸) and manual model building in *Coot* (Emsley *et al.*, 2010 ▸) improved the phases. Further iterative cycles were performed in *phenix.refine* using an intensity-based maximum-likelihood target function, including TLS and NCS refinement with automated group determination as implemented in *phenix.refine*. Strong NCS restraints were applied throughout refinement, leading to a relatively high value for *R*_work_ but a comparably small difference between *R*_work_ and *R*_free_. Because of the low resolution, we assumed that all eight chains were identical and chose to include the same number of amino acids. Consequently, rather poor fits for residues 3–4 of some of the chains remain. Refinement statistics are given in Table 4 ▸. Structural homologs were identified using *DALI* (http://ekhidna2.biocenter.helsinki.fi/dali; Holm, 2020 ▸). Macromolecular interfaces were analyzed using the *PISA* server (http://www.ebi.ac.uk/pdbe/prot_int/pistart.html; Krissinel & Henrick, 2007 ▸).

### 2.5. Circular-dichroism spectroscopy   

Protein concentrations were determined spectrophoto­metrically assuming extinction coefficients at 280 nm of 2980 *M* ^−1^ cm^−1^ for *Vc*ParD2 and 15 470 *M* ^−1^ cm^−1^ for *Vc*ParE2-His as determined from the amino-acid sequences using the *ProtParam* tool from the ExPASy server (Gasteiger *et al.*, 2005 ▸). CD spectra were recorded at room temperature on a Jasco J-715 spectropolarimeter at a concentration of 0.15 mg ml^−1^ in 20 m*M* Tris pH 8.0, 150 m*M* NaCl, 1 m*M* TCEP.

### 2.6. Analytical SEC and SEC-MALS   

Analytical SEC experiments were performed using Superdex Increase 200 10/300 and Superdex Increase 75 10/300 columns (GE Healthcare) equilibrated with 20 m*M* Tris pH 8, 500 m*M* NaCl, 1 m*M* TCEP. The Bio-Rad gel-filtration standards (bovine thyroglobulin, 670 kDa; bovine γ-globulin, 158 kDa; chicken ovalbumin, 44 kDa; horse myoglobin, 17 kDa; vitamin B_12_, 1.35 kDa) were used to make a standard curve of the logarithm of the molecular weights of the standards as a function of their elution volumes.

Multi-angle light-scattering experiments coupled to SEC (SEC-MALS) were performed using a HPLC system (Waters) connected inline with a miniDAWN TREOS II (Wyatt Technology) light-scattering detector (using three angles) followed by a Shodex refractive-index detector (RI-501). A Shodex K402.5-4F SEC column was connected to the SEC-MALS system and equilibrated with 2–3 column volumes of the corresponding running buffer. A dilution series of *Vc*ParD2 samples from 18 to 0.1 mg ml^−1^ was prepared in the same buffer. 10 µl was injected for each dilution. A BSA sample at 1 mg ml^−1^ was used as a standard for calibration. The data were processed, and consequently the molar mass was determined, using the *ASTRA* 7.1.4 software.

### 2.7. Native mass spectrometry (MS)   

For native MS, ParD2 was transferred into different concentrations of ammonium acetate (50, 150 and 500 m*M*) at different pH values (8.0 and 5.6) using Biospin buffer-exchange columns (Bio-Rad, Temse, Belgium). After buffer exchange, the concentration of the protein in the various buffer conditions was determined using a NanoDrop P2000 spectrophotometer (Thermo Scientific, Waltham, Massachusetts, USA).

For each buffer condition, samples were introduced into the mass spectrometer at *Vc*ParD2 concentrations of 0.1, 1.0 and 2.5 mg ml^−1^. Nano-electrospray ionization (ESI) was performed using 3–4 µl of sample loaded into home-made gold-coated borosilicate glass capillaries, with spray voltages applied in the range +1.5–2.0 kV. The spectra were recorded on an ion mobility-enabled time-of-flight mass spectrometer (Synapt G2 HDMS, Waters, Wilmslow, UK). To achieve gentle, native-like conditions, the instrument parameters were carefully optimized in order to avoid ion activation and to preserve the higher order structure of *Vc*ParD2 in the mass spectrometer. The optimized values for *Vc*ParD2 are sampling cone, 50 V; extraction cone, 2 V; trap collision energy, 20 V; trap DC bias, 45 V; transfer collision energy, 4 V. The pressure in the source region (backing) and in the trap cell (collision gas) were 5.5 × 10^−2^ and 3.1 × 10^−2^ mbar, respectively. Spectra were externally calibrated using a 10 mg ml^−1^ solution of caesium iodide. Analyses of the acquired spectra were performed using *MassLynx* version 4.1 (Waters, Wilmslow, UK). Native MS spectra were smoothed (to an extent depending on the size of the complexes) and additionally centred to calculate the molecular weights.

### 2.8. Small-angle X-ray scattering   

SAXS data were collected on the SWING beamline at SOLEIL in HPLC mode using an Agilent BioSEC 3-300 column at a protein concentration of 12 mg ml^−1^ in 20 m*M* Tris pH 8.0, 150 m*M* NaCl, 1 m*M* TCEP at 19°C. Protein samples were briefly spun down before loading onto the SEC column. 50 µl *Vc*ParD2 was injected onto the column at a constant flow rate of 0.2 ml min^−1^. The final scattering curve (after buffer subtraction) was generated for each sample after a range of scattering curves around the peak (with equivalent *R*_g_ values) had been normalized and averaged. The *R*_g_ values were derived from the Guinier approximation at small *q* values, while the *I*(0) parameter was estimated by extrapolation to *q* = 0 using the *ATSAS* suite (Manalastas-Cantos *et al.*, 2021 ▸). Molecular weights were determined by the Bayesian estimation implemented in *PRIMUS* from the *ATSAS* suite.

All simulations were performed in *Xplor-NIH* version 2.49 (Schwieters *et al.*, 2018 ▸), starting from the hexadecameric X-ray structure of *Vc*ParD2 solved in this work. Residues that were not resolved in the X-ray structure, including the disordered C-terminal tail, were added in *Xplor-NIH* using the standard topologies for individual amino acids, followed by minimization of the energy function consisting of the standard geometric (bonds, angles, dihedrals and impropers) and steric (van der Waals) terms.

For refinement against the experimental SAXS data, the positions of the structured protein regions were kept fixed, while the disordered protein termini (including residues 1–4 and 48–81) were given full degrees of freedom. The computational protocol comprised an initial simulated-annealing step followed by side-chain energy minimization as described previously (Schwieters & Clore, 2014 ▸). In addition to the standard geometric and steric terms, the energy function included a knowledge-based dihedral angle potential and a SAXS energy term incorporating the experimental data (Schwieters & Clore, 2014 ▸). Truncated SAXS curves (*q* < 0.5 Å^−1^) were used as the sole experimental input.

In each refinement run, 100 structures were calculated and the ten lowest-energy solutions representing the best agreement with the experimental data were retained for subsequent analysis. The agreement between the experimental and calculated SAXS curves (obtained with the *calcSAXS* helper program, which is part of the *Xplor-NIH* package) was assessed by calculating χ^2^ as 

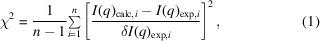

where *I*(*q*)_calc,*i*_ and *I*(*q*)_exp,*i*_ are the scattering intensities at a given *q* for the calculated and experimental SAXS curves, δ*I*(*q*)_exp,*i*_ is an expethemental error on the corresponding *I*(*q*)_exp,*i*_ value and *n* is the number of data points defining the experimental SAXS curve. SAXS experimental details are given in Table 5 ▸.

## 3. Results   

### 3.1. Purification of *Vc*ParD2 and *Vc*ParE2   

We initially attempted to express *Vc*ParD2 in *E. coli* from a pET-28a expression vector that adds a C-terminal His tag followed by a TEV cleavage site. These attempts led to very poor yields, possibly as a consequence of proteolytic degradation of *Vc*ParD2 in the absence of *Vc*ParE2. We therefore altered our strategy and co-expressed *Vc*ParD2 and *Vc*ParE2 to obtain a *Vc*ParD2–*Vc*ParE2 complex. In order to obtain isolated *Vc*ParD2 and *Vc*ParE2, we used an on-column unfolding–refolding protocol similar to those developed previously for Phd/Doc, MazEF and HigBA (Sterckx *et al.*, 2015 ▸). *Vc*ParE2 is His-tagged at its C-terminus and the *Vc*ParD2–*Vc*ParE2 complex was trapped on an Ni–NTA column. Firstly, after extensive washing of the column, the *Vc*ParD2–*Vc*ParE2 complex was eluted and further cleaned using SEC on a Superdex 200 column. This step removes contaminating proteins of lower molecular weight that may otherwise co-migrate with *Vc*ParD2 or *Vc*ParE2 in subsequent steps. Subsequently, the *Vc*ParD2–*Vc*ParE2 complex was reloaded onto the Ni–NTA column. *Vc*ParD2 can then be eluted by applying a GndHCl gradient. This allowed us to prepare *Vc*ParD2 in the absence of an affinity tag or other cloning artefact, ensuring that the quaternary structure is not affected by the presence of a tag. After elution of *Vc*ParD2, the protein was refolded by dialysis. The refolded protein was then further polished by SEC on a Superdex 200 column (Supplementary Fig. S1).

Our method has the additional advantage that *Vc*ParE2 can also be obtained as, like most other TA toxins, *Vc*ParE2 cannot be expressed in the absence of its cognate antitoxin. The *Vc*ParE that remains trapped was refolded on the Ni–NTA column in a three-step procedure that consists of washing with (i) 20 m*M* Tris pH 8.0, 25 m*M* NaCl, 5% glycerol, (ii) 20 m*M* Tris pH 8.0, 150 m*M* NaCl, 5% glycerol and (iii) 20 m*M* Tris pH 8.0, 250 m*M* NaCl, 2 m*M* β-mercaptoethanol. *Vc*ParE2 was subsequently eluted using a step gradient of imidazole. The resulting protein was then applied onto a Superdex 75 column to obtain a sample that is pure on SDS–PAGE.

### 3.2. *Vc*ParD2 is a well folded DNA-binding protein   

**Figure 1:**
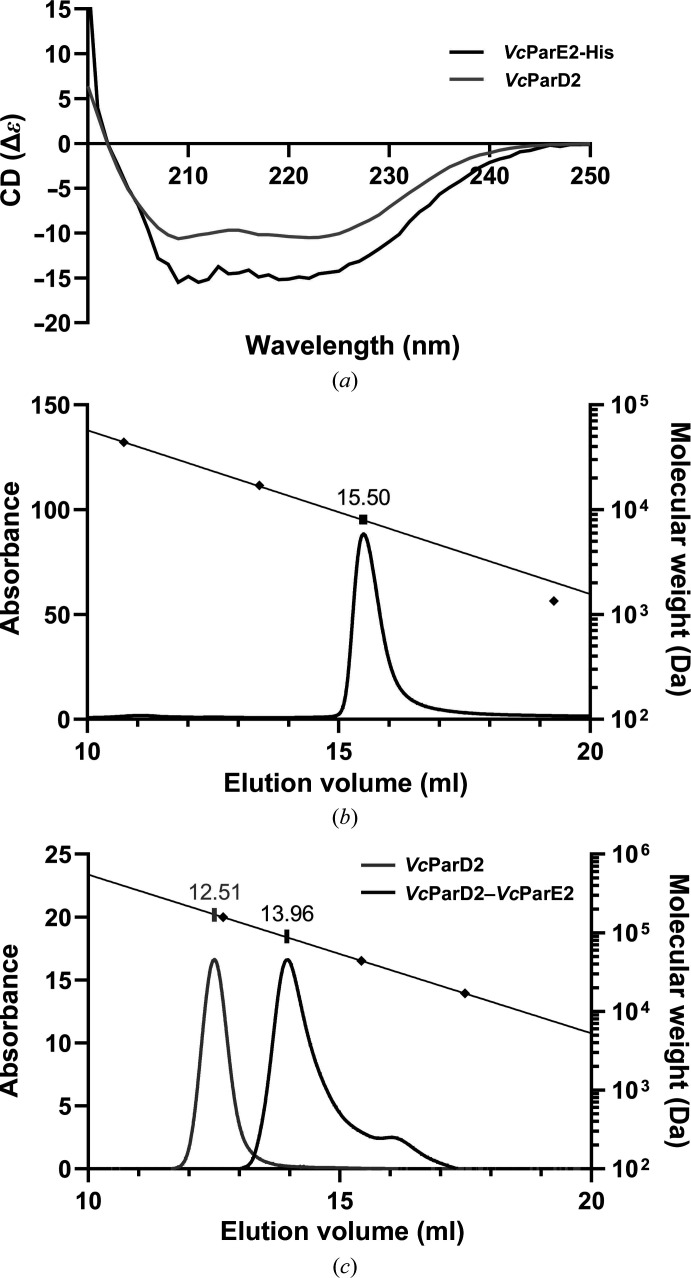
*Vc*ParE2 and *Vc*ParD2 are monodisperse, folded proteins. (*a*) CD spectra of 0.15 mg ml^−1^ *Vc*ParD2 (grey) and *Vc*ParE2-His (black) in 20 m*M* Tris pH 8.0, 150 m*M* NaCl, 1 m*M* TCEP. (*b*) Analytical SEC elution profile of 0.15 mg ml^−1^ *Vc*ParE2 in 20 m*M* Tris pH 8.0, 150 m*M* NaCl, 1 m*M* TCEP on a Superdex Increase 75 10/300 column. The elution volumes of the molecular-weight standards (chicken ovalbumin, 44 kDa; horse myoglobin, 17 kDa; vitamin B_12_, 1.35 kDa) are shown as diamonds together with a linear regression (black line). (*c*) Analytical SEC elution profile of 0.1 mg ml^−1^ *Vc*ParD2 and the *Vc*ParD2–*Vc*ParE2 complex in 20 m*M* Tris pH 8.0, 150 m*M* NaCl, 1 m*M* TCEP on a Superdex Increase 200 10/300 column. The elution volumes of the molecular-weight standards (bovine γ-globulin, 158 kDa; chicken ovalbumin, 44 kDa; horse myoglobin, 17 kDa) are shown as diamonds together with a linear regression (black line).

Both *Vc*ParE2 and *Vc*ParD2 show CD spectra that are compatible with folded proteins (Fig. 1 ▸ *a*). *Vc*ParD2 is almost exclusively composed of α-helices, while *Vc*ParE2 contains both α-helices and β-sheets. The SEC profile of *Vc*ParE2 is compatible with a globular monomer (Fig. 1 ▸ *b*). *Vc*ParD2 nevertheless elutes at an apparent molecular weight of 170 kDa, which is much higher than the 18 kDa expected for a dimer (Fig. 1 ▸ *c*). While the presence of an IDP region will lead to aberrant migration in SEC, this deviation seems to be too large to be solely explainable by this phenomenon and a higher oligomer is likely to be formed. Both elution profiles are nevertheless indicative of monodisperse samples and together with the CD spectra suggest correctly folded proteins.

It is also of interest to compare the molecular weight of *Vc*ParD2 with that of the co-expressed *Vc*ParD2–*Vc*ParE2 complex. The latter elutes from a Superdex Increase 200 10/300 column at an apparent molecular weight of 96 kDa, which is significantly smaller than the corresponding value for the *Vc*ParD2 oligomer (Fig. 1 ▸ *c*). This indicates that *Vc*ParE2 binding at least partially disrupts the *Vc*ParD2 oligomer. The resulting complex is still larger than the corresponding complex from *C. crescentus* (44 kDa; Dalton & Crosson, 2010 ▸).

**Figure 2:**
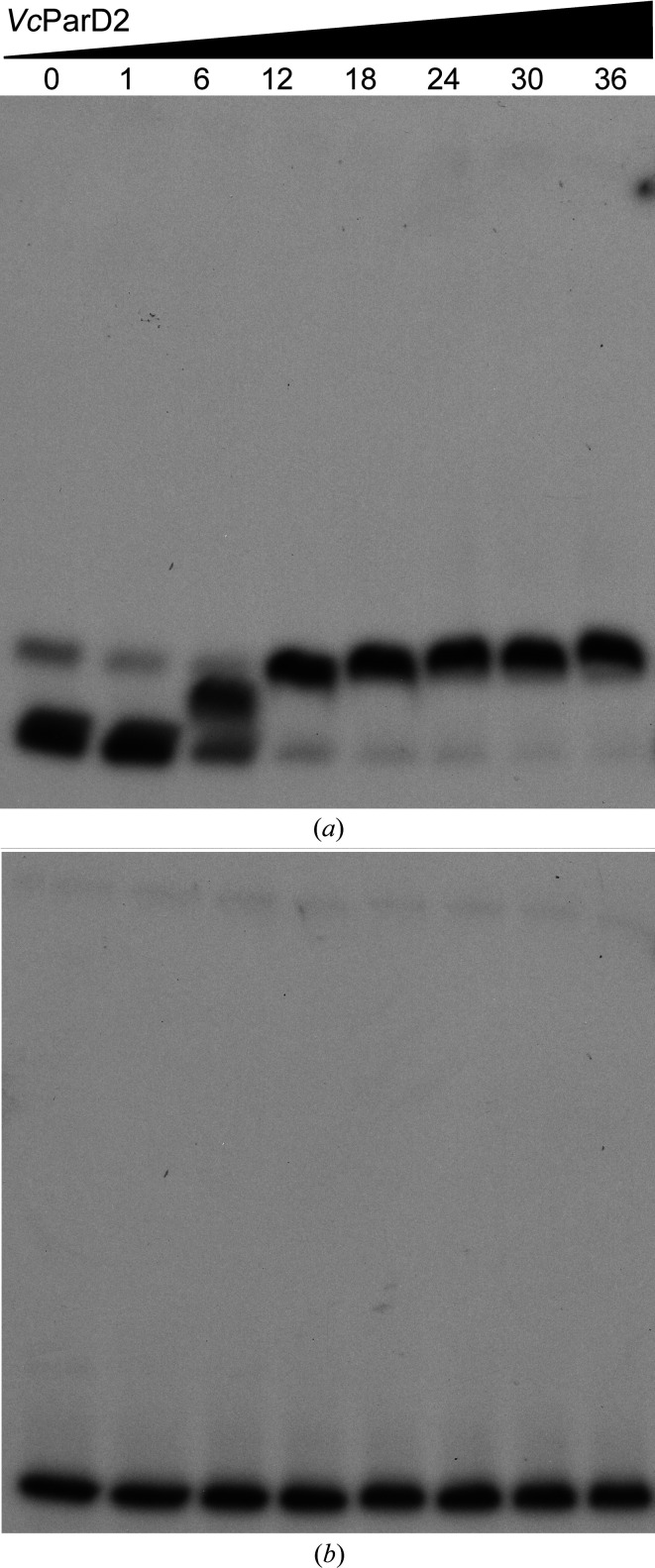
*Vc*ParD2 is a specific DNA-binding protein. (*a*) EMSA experiment with *Vc*ParD2 titrated against a 151 bp DNA fragment upstream of the ATG start codon of ParD2. *Vc*ParD2 concentrations (in monomer equivalents) are given at the top in micromolar units. Binding is observed from 6 µ*M* onwards and involves at least two discrete steps. (*b*) Equivalent EMSA using a 207 bp fragment derived from the operator sequence of the *C. metallidurans psrQ2* gene. The *Vc*ParD2 concentrations used were identical to those in (*a*). No binding was observed for this fragment in the concentration range tested.

In analogy to ParD from plasmid RK2 (*RK*ParD; Roberts *et al.*, 1993 ▸) and ParD2 from *M. tuberculosis* (Gupta *et al.*, 2016 ▸), we predicted *Vc*ParD2 to be a DNA-binding protein that represses the *parDE2* operon. In order to investigate this hypothesis, EMSA experiments were performed using a 151 bp fragment upstream of the translation start of *parD2* and a 207 bp control fragment derived from the operator sequence of the *Cupriavidus metallidurans psrQ2* gene (Ali *et al.*, 2020 ▸). Clear binding can be observed of *Vc*ParD2 to its own operator region, while no binding is observed to the *psrQ2* operator sequence (Fig. 2 ▸). DNA binding therefore appears to be specific and the experiment thus further validates the functionality of our *Vc*ParD2 preparation. With a protein concentration of 6 µ*M* or higher required for binding, this not only corresponds to the same order of magnitude of binding strength as is typical for many bacterial repressors, including *M. tuberculosis* ParD2 (*Mt*ParD2; Gupta *et al.*, 2016 ▸), but also indicates that *Vc*ParD2 is active as a DNA-binding protein in its oligomeric state (see below), further emphasizing the relevance of our structure.

### 3.3. The *Vc*ParD2 monomer and dimer   

The *Vc*ParD2 sample formed crystals that diffracted to 3.1 Å resolution only after three months. The structure was determined by molecular replacement using the dimer of the N-terminal domain of *C. crescentus* ParD2 (*Cc*ParD2; Dalton & Crosson, 2010 ▸), which shows 63% sequence identity to *Vc*ParD2 (54% for the complete protein chain), as a search model. The structure was refined to an *R*_work_ of 0.2709 and an *R*_free_ of 0.2988 (Table 4 ▸). The asymmetric unit contained four dimers of the N-terminal domain of *Vc*ParD2 and the corresponding models encompass residues Lys3–Arg51. The N-terminal two and C-terminal 32 residues are not visible in the electron-density map.

Although we did not succeed in determining the length of the polypeptide present in the crystal, it is highly unlikely that eight intact *Vc*ParD2 chains are present in the asymmetric unit. The solvent content calculated based on the visible part of the *Vc*ParD2 chains is 53%, which is a very reasonable value. In the case of intact 81-amino-acid *Vc*ParD2 chains, this would decrease to 22%. Although such a low solvent content is not entirely impossible, the estimated probability based on the distribution of *V*_M_ values in known crystal structures (Kantardjieff & Rupp, 2003 ▸) is below 1%, and in such a case the crowded environment would be expected to induce structure in the IDP region, as is observed for *M. tuberculosis* YefM (Kumar *et al.*, 2008 ▸) and in part for bacteriophage P1 Phd (Garcia-Pino *et al.*, 2010 ▸). Indeed, degradation of the IDP regions of antitoxins during crystallization has been observed before (Hadži *et al.*, 2017 ▸). Furthermore, limited proteolysis experiments using trypsin, subtilisin and proteinase K showed the appearance of faster moving fragments (one or two closely migrating bands near the buffer front; Supplementary Fig. S2), which further strengthens the hypothesis that the species we have crystallized may have originated via slow (over months) noncontrolled proteolysis.

**Figure 3:**
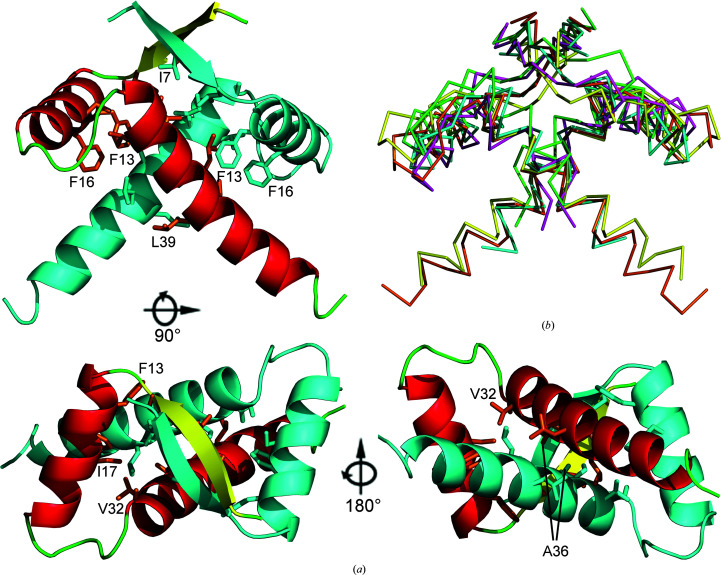
Crystal structure of *Vc*ParD2. (*a*) Cartoon representation of the *Vc*ParD2 dimer (residues 3–51) in three orientations. One monomer is coloured according to secondary structure (β-strand, yellow; α-helices, red; loop regions, green). The second monomer is coloured cyan. (*b*) Superposition of the *Vc*ParD2_N_ dimer (orange) with the dimers of other RHH-type transcription factors: *RK*ParD (green), *Cc*PArD (purple), *Mo*ParD3 (yellow) and *Sa*CopG (cyan).

As expected, the *Vc*ParD2 N-terminal domain (*Vc*ParD2_N_) adopts a ribbon–helix–helix DNA-binding fold (Fig. 3 ▸ *a*), with a C^α^ r.m.s.d. of 0.7 Å on 47 equivalent residues to *Cc*ParD2_N_ (Dalton & Crosson, 2010 ▸; PDB entry 3kxe). Its topology consists of four helices in an open array of two hairpins, as often found in bacterial and phage repressors. In a *DALI* search, a number of additional structurally related transcription factors were identified that not only include the expected N-terminal domain of *M. opportunistum* ParD3, but also CopA, the antitoxin from the newly identified type II TA module ParE_SO_–CopA_SO_ from *Shewanella oneidensis*, the N-terminal domain of the antitoxin AtaR from the *E. coli* AtaT–AtaR TA pair and the non-TA-related 45-residue long transcriptional repressor CopG from *S. agalactiae*, as well as the Arc repressor from *Salmonella* bacteriophage P22 (Table 6 ▸ and Supplementary Fig. S3). Plasmid RK2 ParD, with only 8% sequence identity, only shows up as a low-ranking hit in this search.

Like other members of the MetJ/CopG/Arc family, including its homologs from plasmid RK2, *M. opportunistum* and *C. crescentus*, the *Vc*ParD2 monomers associate into a conserved dimer (Figs. 3 ▸ *a* and 3 ▸ *b*). Dimer formation buries about 1240 Å^2^ of mainly hydrophobic surface and creates the hydrophobic core of the protein. According to the *P*(Δ^i^ *G*) value of 0.37 provided by the *PISA* server (Krissinel & Henrick, 2007 ▸), the contact surfaces are somewhat more hydrophobic that the average surface of a soluble protein, which is in agreement with a dimer that would be stable in solution. Hydrophobic contacts involve contributions from most nonpolar side chains: Ile7 and Leu9 at the end of the N-terminal β-strand, Phe13 and Phe16 in helix α1 and Ile17, Ala29, Val32, Ile33, Ala36, Leu37, Leu39 and Leu40 in helix α2. Most notable is the close contact between the α213 helices of both chains, in which Ala36 seems to be essential, with any other residue except for glycine at this position resulting in steric crowding. Further stabilization arises via hydrogen bonds, most importantly via pairing of the N-terminal β-strands.

### 3.4. Higher order structure in the crystal   

**Figure 4:**
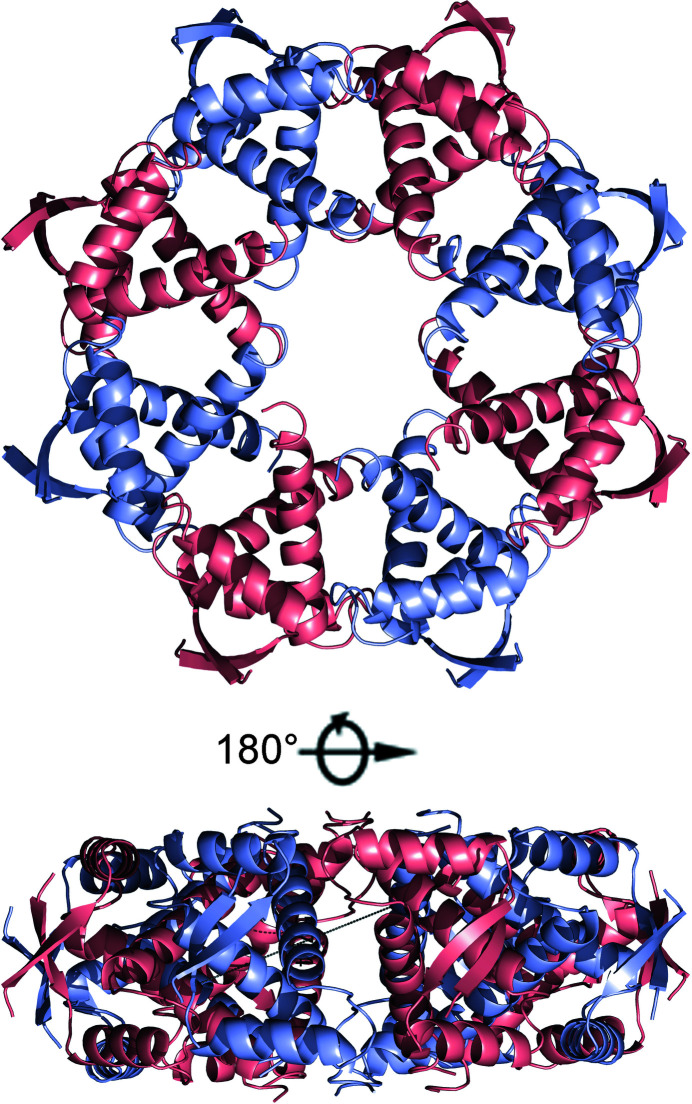
Higher order oligomeric structure of VcParD2_N_. Two orientations of a cartoon representation of the hexadecamer of *Vc*ParD2 as seen in the crystal. *Vc*ParD2 dimers are alternately coloured pink and blue.

**Figure 5:**
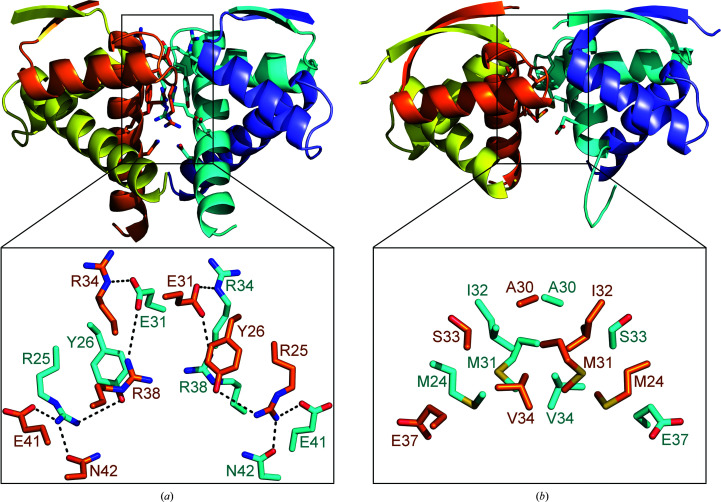
Inter-dimer interface. (*a*) Cartoon representation of the *Vc*ParD2 tetramer. Each chain is coloured differently. Side chains involved in inter-dimer contacts are shown in stick representation and are shown enlarged at the bottom. The contacts are dominated by hydrogen bonds and salt bridges. (*b*) Cartoon representation of the *Sa*CopG tetramer as found in the *Sa*CopG–DNA complex and shown in a similar orientation as *Vc*ParD2. The details of the interacting side chains are again shown enlarged at the bottom. The inter-dimer contacts almost exclusively involve hydrophobic van de Waals interactions.

In the crystal, the *Vc*ParD2_N_ dimers are found in a circular arrangement. This results in a torus consisting of eight *Vc*ParD2_2_ dimers (Fig. 4 ▸). The interface formed by adjacent *Vc*ParD2_2_ dimers buries roughly 600 Å^2^. This interface is highly hydrophilic [*PISA* *P*(Δ^i^ *G*) value of 0.91] and is dominated by hydrogen bonds and salt bridges, and as such deviates from typical stable oligomerization interfaces (Fig. 5 ▸ *a*). The interface is formed by seven amino-acid side chains: Arg25, Tyr26, Glu31, Arg34, Arg38, Glu41 and Asn42. These side chains are mostly involved in inter-side-chain hydrogen bonds or salt bridges (between Glu41 and Arg25 and between Glu31 and Arg34 as well as Arg38) and cation–π stacking between Arg38 and Tyr26. The only hydrogen bond involving a main-chain atom is between the main-chain carbonyl of Arg25 and the side chain of Arg34. Also of interest is the close contact between the side chains of Arg38 in both chains, with their NH2 atoms being only 3.1 Å apart. This, together with the dominance of side chain–side chain interactions (which are entropically less favourable than main chain–main chain interactions), would normally lead us to conclude that this interface only corresponds to a packing contact and is unlikely to exist in solution.

The amino acids contributing to this interface are retained in *Cc*ParD, which has previously been shown to be a dimer in solution (Dalton & Crosson, 2010 ▸). The only substitution among the residues involved in the inter-dimer interface is Asn42, which is Glu43 in *Cc*ParD. This substitution forces Glu42 of *Cc*ParD into a different conformation to the equivalent Glu41 in *Vc*ParD2 in order to separate the two negative charges. This substitution would also result in an interface with a net negative charge of −2. Another potentially relevant difference between *Vc*ParD2 and *Cc*ParD is the reorientation of Arg38 (Arg39 in *Cc*ParD), which now interacts with Glu43 of *Cc*ParD. This further results in an ‘inwards’ movement of Arg26 of *Cc*ParD (Arg25 of *Vc*ParD2) to fill the space vacated by Arg39. The resulting theoretical interface would then be destabilized by close contacts between the positively charged side chains of Arg26 and Arg39 in the adjacent polypeptide chains.

The hole at the centre of the torus has a diameter of about 16 Å. The C-termini of the 16 *Vc*ParD2 N-terminal domains point outwards from the middle of the side surface of the torus. If this arrangement were composed of intact *Vc*ParD2 chains, the C-terminal IDP regions (residues Leu52–Arg81) would be forced to adopt a fan-shaped ensemble that is limited in its conformational variability. In contrast, in *RK*ParD as well as in CcdA the C-terminal IDP ensemble adopts a wide range of conformations, many of them sterically incompatible with the oligomerization of the *Vc*ParD2 N-terminal domains into the torus observed in the crystal (Madl *et al.*, 2006 ▸; Oberer *et al.*, 2007 ▸). It is therefore safe to state that the IDP region would provide an entropic penalty for *Vc*ParD2 oligomerization similar to that observed in the influence of the IDP region of Phd on operator binding (Garcia-Pino *et al.*, 2016 ▸).

The *Vc*ParD2 hexadecamer resembles the crystallographic assembly of *S. agalactiae* CopG, which forms a spiral structure with inter-dimer contact surfaces roughly corresponding to the inter-dimer interfaces seen in the *Vc*ParD2 hexadecamer (Fig. 5 ▸ *b*; Gomis-Rüth *et al.*, 1998 ▸). In contrast to *Vc*ParD2, although similar in size, the oligomerization interface of *Sa*CopG is much more hydrophobic [*PISA* *P*(Δ^i^ *G*) value of 0.38] and is retained in the complex of tetrameric *Sa*CopG with its operator (Gomis-Rüth *et al.*, 1998 ▸; Costa *et al.*, 2001 ▸). Yet in solution, in the absence of a DNA ligand, *Sa*CopG behaves as a dimer.

### 3.5. DNA-binding site   

In general, transcription factors of the ribbon–helix–helix family dock with their N-terminal strands into the major groove of their target DNA. In agreement with this, the N-terminal β-ribbons present a positively charged electrostatic surface on the *Vc*ParD2 dimer that can complement the negative charges of the DNA backbone.

*S. agalactiae* CopG (*Sa*CopG) is the closest structural relative of *Vc*ParD2 for which a structure of a DNA complex is available. *Sa*CopG makes base-pair-specific contacts via the side chains of Arg4 and Thr6, while hydrogen bonds to the DNA backbone are provided by the side chains of Thr8, Lys28 and Ser29 (Gomis-Rüth *et al.*, 1998 ▸; PDB entry 1b01). In *Vc*ParD2, the corresponding residues are Asn4, Ser6, Thr8, Ser29 and Ser31, indicating potential differences in DNA specificity between the two proteins (Supplementary Fig. 3*a* ). Unfortunately, the target sequence of *Vc*ParD2 currently remains unknown.

**Figure 6:**
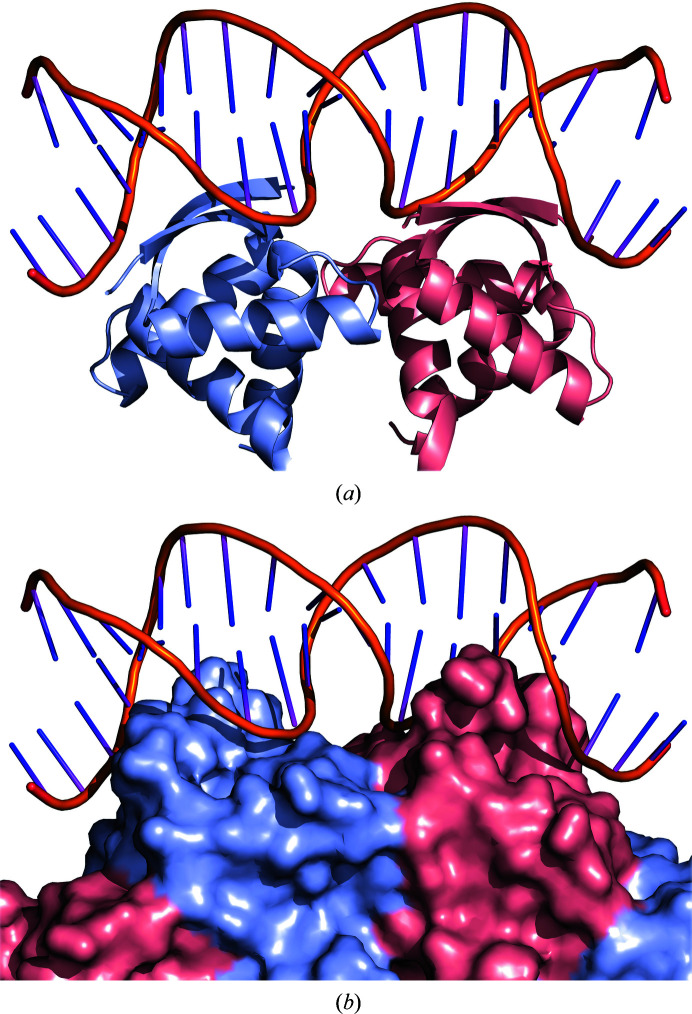
DNA-binding site. (*a*) Complex of *Sa*CopG with an 18 bp DNA fragment. (*b*) Model of *Vc*ParD2 (surface representation as in Fig. 3 ▸ *a*) in complex with DNA. The *Sa*CopG tetramer was superimposed on two adjacent dimers of *Vc*ParD2. The DNA fragment bound to *Sa*CopG fits onto the surface of *Vc*ParD2 with the N-terminal β-strands inserted into the major groove of the DNA.

*Sa*CopG binds its operator in a chain-like fashion. Several dimers position themselves next to each other in a similar way to that seen in the crystal packing of the free *Sa*CopG structure, with the DNA wrapping around the protein helix (Costa *et al.*, 2001 ▸). In contrast to *Sa*CopG, *Vc*ParD2 does not associate into a helical structure, but forms a closed circle. The dimer–dimer association of *Vc*ParD2 is nevertheless very similar to that of *Sa*CopG bound to DNA (Fig. 6 ▸ *a*), suggesting that crystal packing may also reflect associations occurring on the DNA here. Indeed, on the toroidal surface of the *Vc*ParD2 crystallographic hexadecamer, the N-terminal β-strands stick out as a set of eight parallel and equally spaced ridges that lead to a cogwheel-like arrangement (Figs. 3 ▸ and 5 ▸). Fig. 6 ▸(*b*) shows the positioning of an 18 bp fragment with a two-nucleotide overhang taken from an *Sa*CopG–DNA complex and covering two *Sa*CopG dimers on the surface of the *Vc*ParD2 oligomer (Fig. 6 ▸ *a*). When superimposed on *Vc*ParD2, the helical axis of the DNA is tangential to the cogwheel surface, and the subsequent β-strand ridges are spaced so as to make it possible for a double-stranded DNA to wrap around the cogwheel. Thus, it is likely that the oligomerization mode of *Vc*ParD2 may reflect how it interacts with its operator.

### 3.6. Oligomerization in solution   

Despite the fact that there are several arguments that disfavour the existence of the crystalline oligomeric assembly in solution, during the purification of *Vc*ParD2 we observed that it elutes from a Superdex Increase 200 10/300 SEC column at an appreciably higher molecular weight than is expected for a simple dimer, even if we take into account the fact that the IDP region would substantially increase its hydrodynamic radius. To evaluate the relevance of the oligomer seen in the crystal, we determined the oligomeric state of *Vc*ParD2 in solution using SEC-MALS and native mass spectrometry.

**Figure 7:**
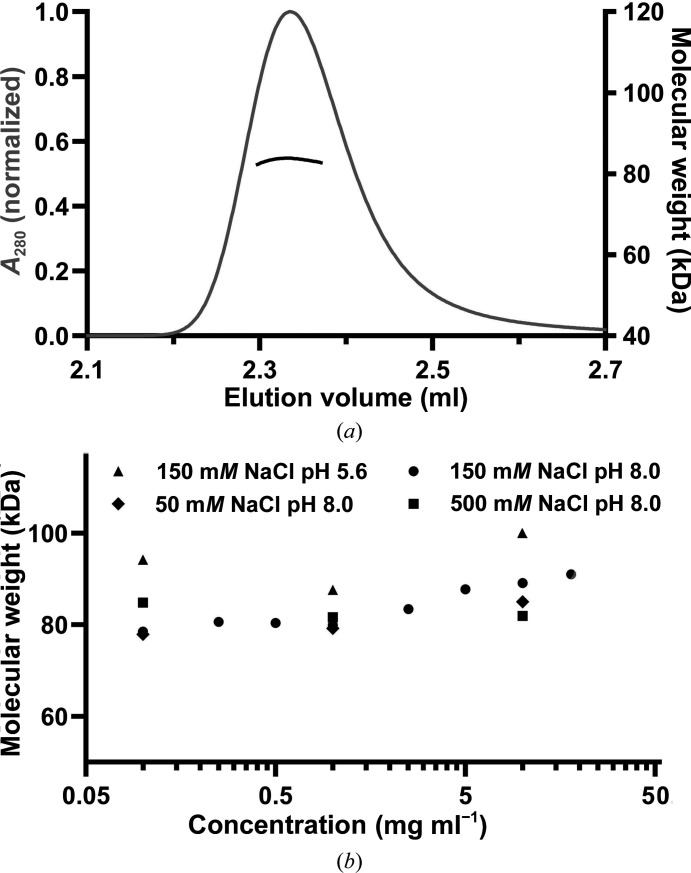
SEC-MALS. (*a*) A typical SEC-MALS run (10 mg ml^−1^ *Vc*ParD2, 20 m*M* Tris pH 8.0, 150 m*M* NaCl, 1 m*M* TCEP). The observed elution peak and plateau for molecular mass is representative of all conditions tested. (*b*) MALS-derived molecular weights as a function of concentration and experimental conditions. No clear dependence on concentration or ionic strength is observed. The molecular-mass values at pH 5.6 are nevertheless systematically higher than for the measurements at pH 8.0.

To obtain a better estimate of the true oligomeric state in solution and how it may be affected by the concentration, we turned to SEC-MALS. A concentration series of *Vc*ParD2 ranging from 18 to 0.1 mg ml^−1^ was injected into a Shodex KW402.5-4F column. At all concentrations, a single peak was observed at an elution volume indicating a higher order oligomer (Fig. 7 ▸, Supplementary Table S1, Supplementary Fig. S4). The molecular weight determined for the protein eluting in this peak ranges from 100.0 ± 0.6 kDa for the highest concentration used to 78.5 ± 0.6 kDa for the lowest concentration used. No additional peaks were observed that could be attributed to a monomer or dimer. Given that the theoretical molecular weight for a *Vc*ParD2 dimer is 17 912 Da and that it can be assumed that the oligomeric state of *Vc*ParD2 is a multiple of a dimer, the SEC-MALS data indicate the presence of mainly decamers, which are likely to be in equilibrium with dodecamers and octamers.

Little dependence on concentration or ionic strength was observed, although lower concentrations tend to give somewhat lower average molecular weights. Similarly, a lower pH leads to a somewhat higher average molecular weight. Most important, however, is that even at the highest concentration used the experimental molecular weight is significantly less than that of a hexadecamer (as suggested from the crystal structure). This deviation from the expected molecular weight is not due to degradation of the C-termini, as nESI-TOF mass spectra of the protein sample after performing the SEC-MALS experiments showed the protein to be intact, with no signs of degradation.

**Figure 8:**
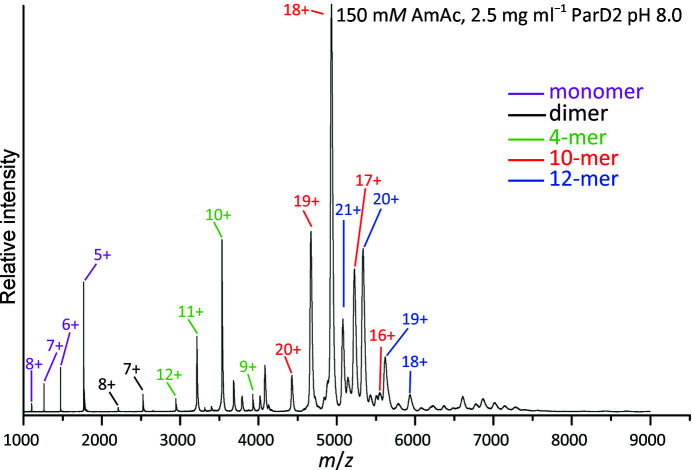
Native mass spectrometry. The native mass spectrum of *Vc*ParD2 (2.5 mg ml^−1^) in 150 m*M* ammonium acetate pH 8.0 is shown with the major peaks labelled according to their charge states. The major species present are decamers and dodecamers, with smaller amounts of monomers, dimers and tetramers also being observed.

To further understand the true nature of the higher order complexes that are present in solution, we performed native mass spectrometry (Fig. 8 ▸, Supplementary Fig. S5). These data nicely mirror the results obtained using SEC-MALS and show that *Vc*ParD2 is predominantly present as a mixture of decamers and dodecamers in solution, with tetramers as a third species. Similar to the observations using SEC-MALS, a lower pH favours the dodecameric assembly. At the lowest concentration (0.1 mg ml^−1^), some monomers, tetramers and a very small amount of dimers can nevertheless also be seen, but decamers and dodecamers still dominate, indicating that these are indeed stable species. The stability of the decamers and dodecamers, particularly at high ionic strength, is surprising given the electrostatic nature of the inter-dimer contact interface, as also is the lack of substantial amounts of tetramers, hexamers or octamers. We do not observe the presence of tetradecamers or the hexadecamer that would be expected from the crystal structure.

### 3.7. Solution SAXS model of the *Vc*ParD2 oligomer   

**Figure 9:**
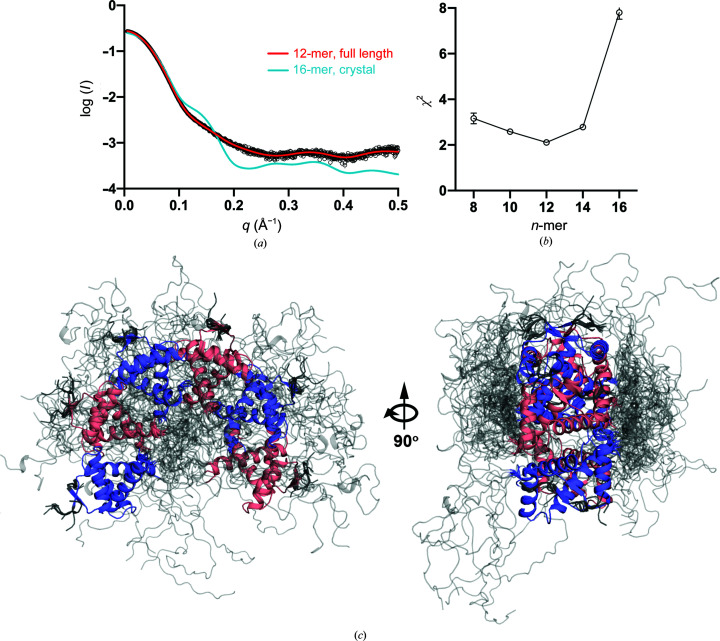
Small-angle X-ray scattering. (*a*) Experimental SAXS data (black) overlaid with the theoretical scattering curve for a *Vc*ParD2 dodecamer averaged over the ten best-fitting conformations (red; χ^2^ = 2.11) and the curve calculated for the hexadecameric crystallographic assembly (cyan). (*b*) χ^2^ values for the best fits of octameric, decameric, dodecameric, tetradecameric and hexadecameric ensembles. A minimum is seen for a dodecameric ensemble. (*c*) Molecular model of the dodecameric ensemble that best represents the scattering data. The folded parts of *Vc*ParD2 dimers are alternately shown in blue and pink. The IDP tails are shown in grey.

In order to obtain a more detailed picture of the oligomeric species in solution, we turned to small-angle X-ray scattering (SAXS). The protein used in this experiment was again confirmed to be intact using nESI-TOF mass spectrometry, and therefore a significant fraction of its polypeptide (27%) is expected to be present in a disordered ensemble. SAXS data collected on the SWING beamline at SOLEIL were of high quality up to a *q* value of 0.5 (Fig. 9 ▸ *a* and Table 5 ▸). The Guinier plot shows linear behaviour (*R* ^2^ = 0.97), rendering a radius of gyration of 33.64 ± 0.34 Å. However, the dimensionless Kratky plot of *Vc*ParD2 does not reveal the disordered nature of the C-terminal regions (Supplementary Fig. S6). Its bell-shaped curve with a maximum around (1.73, 1.1) agrees with the expected values for a globular particle. The *P*(*r*) function shows a nice bell shape and converges smoothly to zero, with a good fit at low *q* angles, indicative of a polydisperse sample (Manalastas-Cantos *et al.*, 2021 ▸).

SAXS data for the *Vc*ParD2 antitoxin indicate a molecular mass of approximately 109 kDa from the Bayesian estimate implemented in the *ATSAS* suite (Manalastas-Cantos *et al.*, 2021 ▸). The oligomeric state suggested by this molecular mass thus corresponds to a dodecamer. The sample used in these experiments was subsequently verified by nESI-TOF mass spectrometry and was shown to consist only of intact chains. In agreement with this observation, the theoretical SAXS curve calculated for the hexadecameric crystallographic assembly does not fit the experimental scattering curve (χ^2^ = 28.16). Equally, the full-length protein in this hexadecameric assembly, with an ensemble in which residues 5–47 are fixed and residues 1–4 and 48–81 are given full torsional degrees of freedom, still provides a poor fit (χ^2^ = 7.80). In this ensemble, the IDP tails tend to plug the central hole, rendering the whole particle more globular. While these solutions are physically possible (there are very few, if any van der Waals clashes, torsion angles are good and local geometry is perfect), they do not make sense biochemically. Entropic considerations make such a structure, with the tails crammed up in the middle, highly unlikely.

We then considered a number of ensembles consisting of full-length *Vc*ParD2 chains in octameric, decameric, dodecameric and tetradecameric arrangements. As seen in Fig. 9 ▸(*b*), the best agreement is obtained for a dodecamer that forms an open fragment of a torus with identical relative orientations of the contacts between adjacent dimers as seen in the hexa­decameric arrangement in the crystal (χ^2^ = 2.11). This ensemble features a realistic spread of tails, with some of them visiting the central inter-domain region (the former central hole) and others flopping about on the periphery (Fig. 9 ▸ *c*). Nevertheless, similar decameric and tetradecameric models also fit reasonably well (χ^2^ = 2.58 and 2.78, respectively) and therefore cannot be excluded. Hybrid models containing increasing fractions of decameric structures added to the dodecameric ensemble do not further improve the fit. In other words, the dodecameric ensemble on its own is sufficient to explain the scattering data and is most likely to be the dominant species present in solution.

## 4. Discussion   

We were able to obtain correctly folded *Vc*ParD2 from the overexpressed *Vc*ParD2–*Vc*ParE2 complex via an on-column unfolding–refolding procedure and showed that the resulting protein interacts specifically with a 151 bp DNA segment upstream of the ATG start codon of the *parD2* gene. Transcription regulation of *parDE* modules has also been investigated for *parDE* on *E. coli* plasmid RK2 and *parDE2* on the chromosome of *M. tuberculosis* H37Rv. For plasmid RK2 *parDE*, the antitoxin *RK*ParD is responsible for auto-repression of the operon, with two *RK*ParD dimers binding to the operator. In contrast to most other TA families, *RK*ParE does not seem to modulate *RK*ParD-mediated repression *in vivo* (Roberts *et al.*, 1993 ▸; Johnson *et al.*, 1996 ▸; Oberer *et al.*, 1999 ▸). In the case of *M. tuberculosis* *parDE2*, *Mt*ParD2 also represses the operon on its own, but the presence of *Mt*ParE2 reduces repression *in vivo* (Gupta *et al.*, 2016 ▸). Previous *in vivo* data from the *V. cholerae* *parDE2* module are in line with those for plasmid RK2 *parDE*, with *Vc*ParE2 not being involved in auto-regulation (Yuan *et al.*, 2011 ▸). Our *in vitro* DNA-binding results are in line with the results for the homologous *parDE* modules located on plasmid RK2 and the *M. tuberculosis* chromosome. The affinity of *Vc*ParD2 is in the low-micromolar range and its higher order oligomeric structure suggests multiple adjacent binding sites, as is also the case for *RK*ParD, although the latter is a dimer in solution.

The folded domains of *Vc*ParD2 form a partial doughnut structure in solution that is stabilized via strong electrostatic interactions. While not recognized as such by the *PISA* server (Krissinel & Henrick, 2007 ▸), this association is stable and is maintained even at low protein concentrations. For over four decades, hydrophobicity, together with complementarity, has been believed to be the major factor stabilizing protein–protein association (Chothia & Janin, 1975 ▸). Contact surfaces in protein oligomers differ from the rest of the subunit surface in that they are enriched in hydrophobic side chains and have a low density of inter-subunit hydrogen bonds (Janin *et al.*, 1988 ▸; Lo Conte *et al.*, 1999 ▸). In small dimeric proteins, the dimer interface often corresponds to the hydrophobic core and dimerization is essential to form a stable structure.

In contrast, high-affinity electrostatic interactions are known between IDPs, for example the disordered complex formed between histone H1 and its chaperone prothymosin-α (Borgia *et al.*, 2018 ▸). Similar poly-electrolytic interactions have also been postulated to drive the formation of membraneless organelles via liquid–liquid phase transitions (Brangwynne *et al.*, 2015 ▸; Schuler *et al.*, 2020 ▸). On the other hand, to find a stable association of globular proteins dominated by side chain–side chain hydrogen bonds and electrostatic inter­actions is highly unusual. To our knowledge, no other examples of such complexes are known, and the *Vc*ParD2 oligomer thus represents an example of a rare class of protein–protein interface. The stability of this oligomer is even more surprising when one considers that the amino acids involved in this interaction are highly conserved in *Cc*ParD from *C. crescentus*, with the only difference being the substitution of an asparagine by a glutamate in *Cc*ParD. The latter would bury two negative charges at the inter-dimer interface, creating a highly unstable situation.

In the crystal, the globular domain of *Vc*ParD2 forms a closed doughnut-shaped structure in the absence of its IDP tail, which most likely degraded in the two months that were required for crystals to appear. In the absence of any cooperativity, one would expect that with identical interactions between all dimers, a complete circle would also be formed in solution. Nevertheless, we were able to demonstrate that the doughnut is incomplete in solution and that the assembly consists of 5–6 *Vc*ParD2 dimers instead of eight. This implies the existence of negative cooperativity that prevents formation of the closed structure. It is likely that this negative cooperativity originates from the IDP tails, which in a full hexadecameric assembly would hinder each other’s freedom and create an entropic barrier that prevents an oligomer larger than a dodecamer from forming. A similar entropic exclusion principle has previously been observed for the binding of Phd to its operator (Garcia-Pino *et al.*, 2016 ▸). Two copies of the Phd dimer need to bind at adjacent sites, but this is prevented due to entropic exclusion of the IDP tails. Only when Doc binds and folds these IDP tails can operator binding proceed with high affinity and the *phd/doc* operon be repressed. This mechanism is further related to the action of entropic bristles, which are IDP tails that can act as solubilizers to prevent aggregation (Santner *et al.*, 2012 ▸), tune prion nucleation (Michiels *et al.*, 2020 ▸) and tune the energy landscape of proteins in general with respect to protein assemblies and ligand binding and association (Keul *et al.*, 2018 ▸; Niemeyer *et al.*, 2020 ▸).

The similarities in higher order association between *Vc*ParD2 and *Sa*CopG from *S. agalactiae* suggest a mechanism for DNA binding. *Vc*ParD2 and *Sa*CopG make higher order contacts via the same spatial surface, although this surface is substantially more hydrophobic in *Sa*CopG. This association in *Sa*CopG leads to an extended DNA-binding surface where multiple *Sa*CopG dimers dock next to each other on the operator. In many TA systems, the antitoxin often binds to two or more binding sites on the operator. In most cases, the toxin increases antitoxin–DNA affinity by bridging adjacent antitoxin dimers (Garcia-Pino *et al.*, 2010 ▸; Vandervelde *et al.*, 2017 ▸; Xue *et al.*, 2020 ▸) or by otherwise stabilizing the DNA-binding assembly of the antitoxin (Bøggild *et al.*, 2012 ▸; Qian *et al.*, 2019 ▸; Jurėnas *et al.*, 2019 ▸). Higher toxin:antitoxin ratios lead to de-repression via the formation of toxin–antitoxin complexes with altered stoichiometry. For antitoxins that only bind to a single site, regulation is less complex and the toxin only serves to weaken operator binding (Brown *et al.*, 2013 ▸; Turnbull & Gerdes, 2017 ▸; Winter *et al.*, 2018 ▸; Manav *et al.*, 2019 ▸). For *parDE* modules, the mechanism of transcription regulation is not known. Early studies of the *parDE* system present on plasmid RK2 indicated that the antitoxin is sufficient to repress the operon and is likely to bind on two adjacent palindromes (Roberts *et al.*, 1993 ▸). Although the operator of the *V. cholerae* *parDE2* operon is not known, the higher order association of *Vc*ParD2 dimers suggests cooperative binding to the DNA in a similar way to that observed for *Sa*CopG. The extent to which the corresponding toxin influences this interaction currently remains unknown.

## Supplementary Material

PDB reference: N-terminal domain of ParD2, 7b22

Supplementary Figures and Table. DOI: 10.1107/S2059798321004873/dw5221sup1.pdf

## Figures and Tables

**Table 1:** Macromolecule-production information

Source organism	*V. cholerae*
DNA source	Commercial gene synthesis by GenScript
Cloning vector	pET-28a
Expression vector	pET-28a
Expression host	*E. coli* BL21 (DE3)
Complete amino-acid sequence of the construct produced	
*Vc*ParD2	MAKNTSITLGEHFDGFITSQIQSGRYGSASEVIRSALRLLENQETKLQSLRQLLIEGEQSGDADYDLDSFINELDSENIR
*Vc*ParE2	MKPFNLTVAAKADLRDIALFTQRRWGKEQRNVYLKQFDDSFWLLAENPDIGKSCDEIREGYRKFPQGSHVIFYQQTGSQQIRVIRILHKSMDVNPIFGAHHHHHH

**Table 2:** Crystallization

Method	Sitting drop
Plate type	96-well Intelli-Plate (Hampton Research)
Temperature (K)	293
Protein concentration (mg ml^−1^)	20
Buffer composition of protein solution	20 m*M* Tris pH 8.0, 150 m*M* NaCl
Composition of reservoir solution	0.2 *M* lithium sulfate, 0.1 *M* MES pH 6.0, 20%(*w*/*v*) PEG 4000
Volume and ratio of drop	0.2 µl, 1:1
Volume of reservoir (µl)	100

**Table 3:** Data collection and processing Values in parentheses are for the outer shell.

Diffraction source	PROXIMA-2A, SOLEIL
Wavelength (Å)	0.9801
Temperature (K)	100
Detector	EIGER X 9M
Crystal-to-detector distance (mm)	404.12
Rotation range per image (°)	0.1
Total rotation range (°)	240
Space group	*C*222_1_
*a*, *b*, *c* (Å)	85.01, 101.70, 107.23
α, β, γ (°)	90, 90, 90
Mosaicity (°)	0.208
Resolution range (Å)	45.95–3.083 (3.193–3.083)
Total No. of reflections	59911 (4033)
No. of unique reflections	8845 (842)
Completeness (%)	99.50 (96.34)
Multiplicity	6.8 (4.8)
〈*I*/σ(*I*)〉	7.36 (0.93)
*R*_r.i.m._	0.1738 (1.411)
Overall *B* factor from Wilson plot (Å^2^)	77.81

**Table 4:** Structure solution and refinement Values in parentheses are for the outer shell.

Resolution range (Å)	45.95–3.08 (3.19–3.08)
Completeness (%)	99.50 (96.34)
σ Cutoff	None
No. of reflections, working set	8845 (842)
No. of reflections, test set	442 (42)
Final *R*_cryst_	0.27 (0.35)
Final *R*_free_	0.29 (0.36)
No. of non-H atoms	2856
R.m.s. deviations	
Bonds (Å)	0.001
Angles (°)	0.30
Average *B* factor (Å^2^)	84.57
Ramachandran plot	
Most favoured (%)	95.74
Allowed (%)	3.99
Disallowed (%)	0.27

**Table:** (*a*) Sample details.

Protein name	ParD2
Organism	*V. cholerae* O1 El Tor strain
Source/entry	Synthetic gene
Sequence	MAKNTSITLGEHFDGFITSQIQSGRYGSASEVIRSALRLLENQETKLQSLRQLLIEGEQSGDADYDLDSFINELDSENIR (UniProt ID P58093)
Extinction coefficient ɛ	2980 *M* ^−1^ cm^−1^ (280 nm)
Partial specific volume \bar \nu (cm^3^ g^−1^)	0.742
Mean scattering contrast \Delta \overline {\rho} (cm^−2^)	2.80 × 10^10^
Molecular mass *M* from chemical composition (Da)	8964.84
SEC-SAXS	
Loading volume/concentration (mg ml^−1^)	12
Injection volume (µl)	50
Flow rate (ml min^−1^)	0.2
Solvent	20 m*M* Tris pH 8, 150 m*M* NaCl, 1 m*M* TCEP

**Table:** (*b*) SAXS data-collection parameters.

Source	SWING beamline, SOLEIL synchrotron
Wavelength (Å)	1.03219
Beam size (µm)	200 × 20 to 500 × 200 (KB), 20 × 20 (micro-focus)
Sample-to-detector distance (m)	2.0
*q*-measurement range (Å^−1^)	0.0054–0.5040
Basis for normalization to constant counts	Data were normalized to the intensity of the transmitted beam and radially averaged; the scattering of the solvent blank was subtracted
Method for monitoring radiation damage	Evaluation of *R*_g_ values per frame during data collection
Exposure time (s)	0.990
Sample configuration	In-line size-exclusion chromatography (SEC) at 0.2 ml min^−1^. Quartz capillary 1.5 mm diameter.
Sample temperature (°C)	19

**Table:** (*c*) Software employed for SAXS data reduction, analysis and interpretation.

SAXS data reduction	*FOXTROT* 3.5.2-3645
Calculation of ɛ from sequence	ExPASy *ProtParam* tool
Basic analyses: Guinier, *P*(*r*), scattering particle volume	*PRIMUS* from the *ATSAS* 2.8 package
Atomic structure modelling	*Xplor-NIH* version 2.49
Modelling of missing sequences from PDB files	*Xplor-NIH* version 2.49
Molecular graphics	*UCSF Chimera*

**Table:** (*d*) Structural parameters.

Guinier analysis	
*I*(0) (cm^−1^)	0.28
*R*_g_ (Å)	32.64
*q*-range (Å^−1^)	0.0001–0.0013
Quality-of-fit parameter	0.97 (legacy estimate implemented in *PRIMUS*/*ATSAS*)
*M* from *I*(0) (ratio to expected value)	109000 Da (1 to the mass of a dodecameric assembly)
*P*(*r*) analysis	
*I*(0) (cm^−1^)	0.28
*R*_g_ (Å)	33.08
*d*_max_ (Å)	139.27
*q*-range (Å^−1^)	0.01–0.23
Quality-of-fit parameter	0.74 (legacy estimate implemented in *PRIMUS*/*ATSAS*)
*M* from *I*(0) (ratio to expected value)	124000 (1.1)
Volume (Å^3^)	127962

**Table:** (*e*) Atomistic modelling.

Method	Simulations in *Xplor-NIH* based on crystallographic coordinates of the hexademeric assembly
*q*-range for fitting (Å^−1^)	0–0.5
χ^2^ value	2.11 (average of the ten best dodecameric models)
Adjustable parameters in the model fit	Side-chain energy minimization, geometric and steric terms, knowledge-based dihedral angle potential included in the energy function
Domain/subunit coordinates and contacts, regions of presumed flexibility	Positions of the structured protein regions were kept fixed, while the disordered protein termini (including residues 1–4 and 48–81) were given full degrees of freedom

**Table:** (*f*) Data and model deposition IDs.

PDB code of crystallographic structure	7b22; PDB files of dodecamer models are available upon request

**Table 6:** Structural homologs of the *Vc*ParD2 N-terminal domain picked up in a *DALI* search

Protein	Organism	PDB code	*DALI Z*-score	R.m.s.d. (Å)	Sequence identity (N-terminal domain) (%)	No. of common C^α^ atoms	Reference
*Cc*ParD	*Caulobacter crescentus*	3kxe	7.5	0.7	63	47	Dalton & Crosson (2010 ▸)
*So*CopA	*Shewanella oneidensis*	6iya	6.2	1.8	20	46	Zhao *et al.* (2019 ▸)
*Mo*ParD3	*Mesorhizobium opportunistum* WSM2075	5ceg	6.1	1.7	17	43	Aakre *et al.* (2015 ▸)
*Sa*CopG	*Streptococcus agalactiae*	2cpg	5.6	1.8	23	43	Gomis-Rüth *et al.* (1998 ▸)
Arc	*Salmonella* bacteriophage P22	1arq	5.4	1.9	7		Bonvin *et al.* (1994 ▸)
*Ec*AtaR	*Escherichia coli*	6ajn, 6gto	5.2	2.1	9	44	Yashiro *et al.* (2019 ▸), Jurėnas *et al.* (2019 ▸)
*RK*ParD	*Escherichia coli* RK2 plasmid	2an7	4.4	2.1	8	33	Oberer *et al.* (2007 ▸)
