# Supplementary material for: Entropic pressure controls the oligomerization of the Vibrio cholerae ParD2 antitoxin
Source: Acta Crystallogr D Struct Biol. 2021 Jun 18;77(Pt 7):904–20. doi: 10.1107/S2059798321004873 (PMC8251345; doi:10.1107/S2059798321004873)
Supplement: Supplementary file 1 [file d-77-00904-sup1.pdf]

STRUCTURAL BIOLOGY

Volume 77 (2021)

Supporting information for article:

Entropic pressure controls the oligomerization of the *Vibrio cholerae* ParD2 antitoxin

Gabriela Garcia-Rodriguez, Yana Girardin, Alexander N. Volkov, Ranjan Kumar Singh, Gopinath Muruganandam, Jeroen Van Dyck, Frank Sobott, Wim Versées, Daniel Charlier and Remy Loris

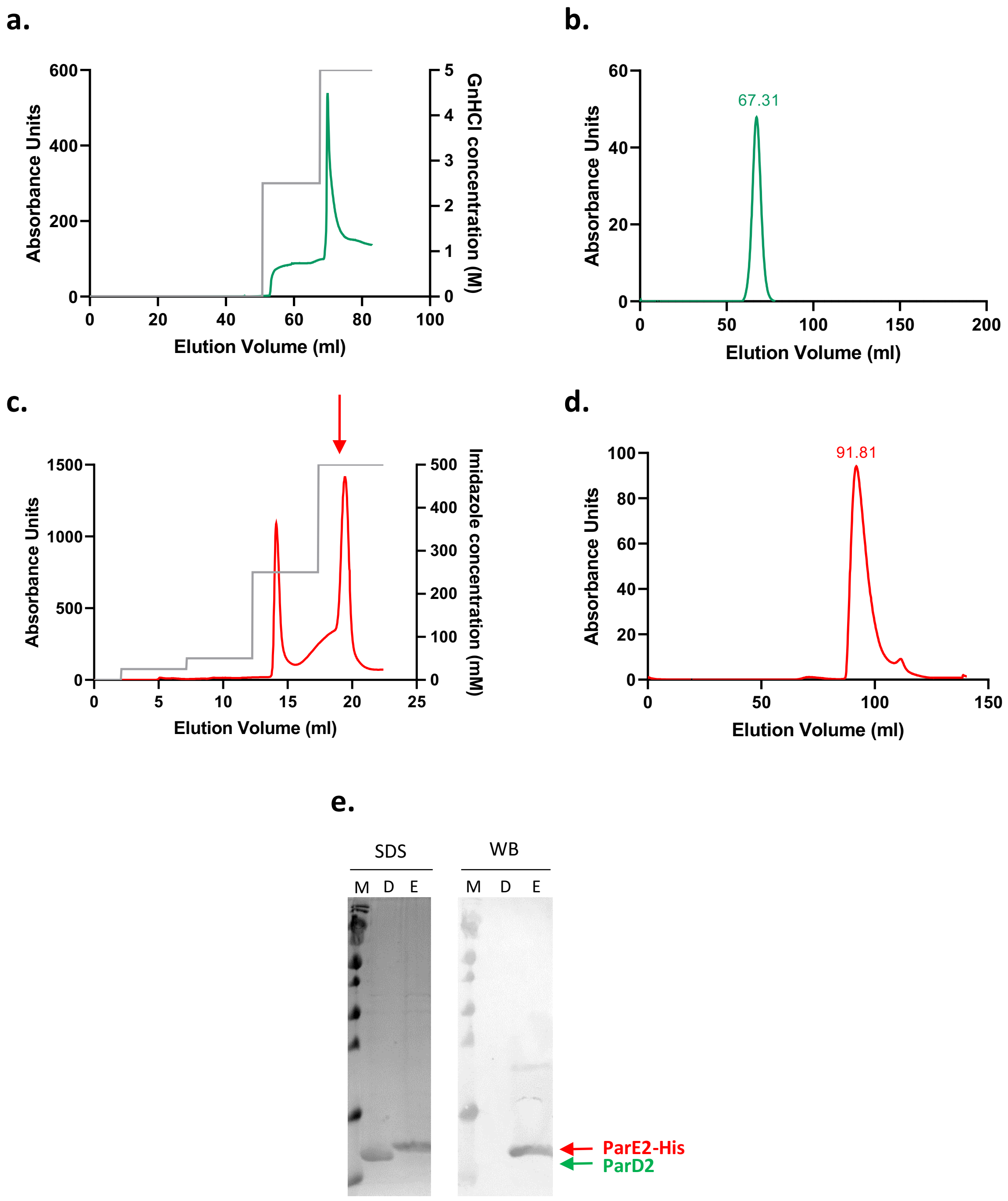

Supplementary Figure S1. Purification of *Vc*ParD2 and *Vc*ParE2. **A.** Denaturant-induced dissociation of the *Vc*ParD2:*Vc*ParE2-His complex, resulting in free *Vc*ParD2 and *Vc*ParE2-His trapped on a Ni-NTA column. *Vc*ParD2 from the HisTrap column with a step-gradient of GnHCl. The thus obtained *Vc*ParD2 is refolded by dialysis in 20 mM Tris pH 8, 150 mM NaCl and concentrated to a volume of 2.5 ml **B.** SEC cleaning of the concentrated *Vc*ParD2 on a Superdex 200 16:60 column equilibrated with 20 mM Tris pH 8, 150 mM NaCl. **C.** Elution of on-column refolded *Vc*ParE2-His from the Ni-NTA column using a step-gradient of imidazole. While the first peak still contains some contaminants, the second peak is essentially pure and is concentrated to a volume of 2.5 ml. **D.** SEC cleaning of the concentrated *Vc*ParE2-His on a Superdex 75 16:60 column equilibrated with 20 mM Tris pH 8, 150 mM NaCl, 1 mM TCEP. **E.** SDS-PAGE and corresponding anti-His-tag Western Blot of the pure *Vc*ParD2 and *Vc*ParE2-His proteins after SEC.

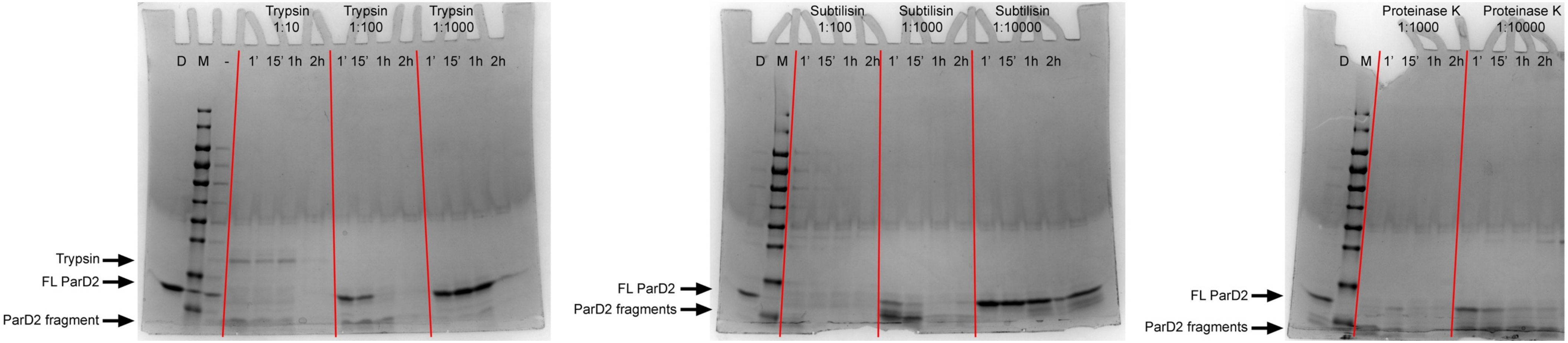

Supplementary Figure S2. Limited proteolysis of *Vc*ParD2. *Vc*ParD2 was subjected to different concentrations of Trypsin, Subtilisin or Proteinase K for 1 min, 15 min, 1 hour or 2 hours. Depending on enzyme concentration and reaction time, a single (Trypsin) or two closely spaced (Subtilisin or Proteinase K) bands are observed resulting from degradation of *Vc*ParD2.

a.

```
     VcParD2  -------LEEEEELLLLHHHHHHHHHHLLLLLLHHHHHHHHHHHHHHHHHLLLLLL----------------------------------------
VcParD2       -----maKNTSITLGEHFDGFITSQIQSGRYGSASEVIRSALRLLENQETKLQSLRqlliegeqsgdadydldsfineldsenir-----------
CcParD  3kxe  ----maskNTSVVLGDHFQAFIDSQVADGRYGSASEVIRAGLRLLEENEAKLAALRAALIEGEESGFIEDFDFDAFIEERSRA-------------
MoParD3 5ceg  --masnvEKMSVAVTPQQAAVMREAVEAGEYATASEIVREAVRDWLAKRELRHDDIRRLRQLWDEGKASGRPEPVDFDALRKEARQKLTEvppngr
RK2ParD 2an7  ------mSRLTIDMTDQQHQSLKALAAL-QKTI-KQYALERLFPGDADADQAWQELKTMLGNRINDGLAGKVSTKSVGEILDEELSGDRA------
SaCopG  1ea4  -----MKKRLTITLSESVLENLEKMAREMGLSK-SAMISVALENYKKGQek---------------------------------------------
EcAtaR  6gto  -msavkkQRIDLRLTDDDKSMIEEAAAISNQSV-SQFMLNSASQRAAEVIEQHRRVilneeswtrvmdalsnppspgeklkraakrlqgm------
SoCopA  6iya  ---mSTIKPVSVKLDADIKARVEHLAETRKRSS-HWMMREAIREYVEREEKREAL-----------------------------------------
P22 Arc 1arq  mkgmskmPQFNLRWPREVLDLVRKVAEEN-GRSVNSEIYQRVMESFKKEGRIGA------------------------------------------
                          : : :       :   : :          :   :
```

b.

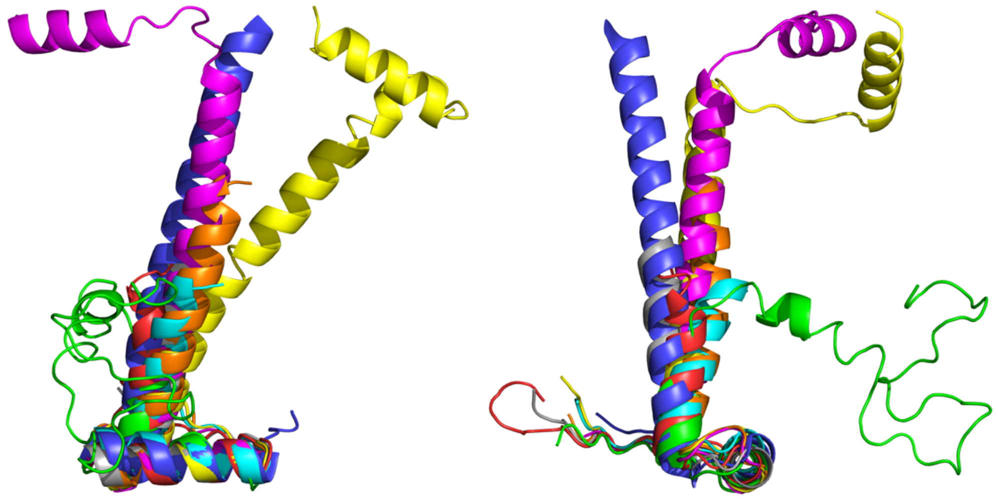

Figure S3. A. Structure-based sequence alignment of *Vc*ParD2 and selected RHH proteins. Residues that are not present in the model (because of disordered in the corresponding crystal structure) are shown in small letters. residues that have structurally equivalent residues in *Vc*ParD2 are shown in bold. Residues highlighted in green form the hydrophobic core of the *Vc*ParD2 dimer while those in cyan mediate the inter-dimer contacts in the higher order oligomer. Residues with side chains known to be involved in DNA recognition are highlighted in yellow. **B. Superposition** of the monomers of *Vc*ParD2 (orange), *Cc*ParD (magenta), *Mo*ParD3 (yellow) *RK2*ParD 2an7 (green), *Sa*CopG (cyan), *Ec*AtaR (blue), *So*CopA (grey) and P22 Arc (red).

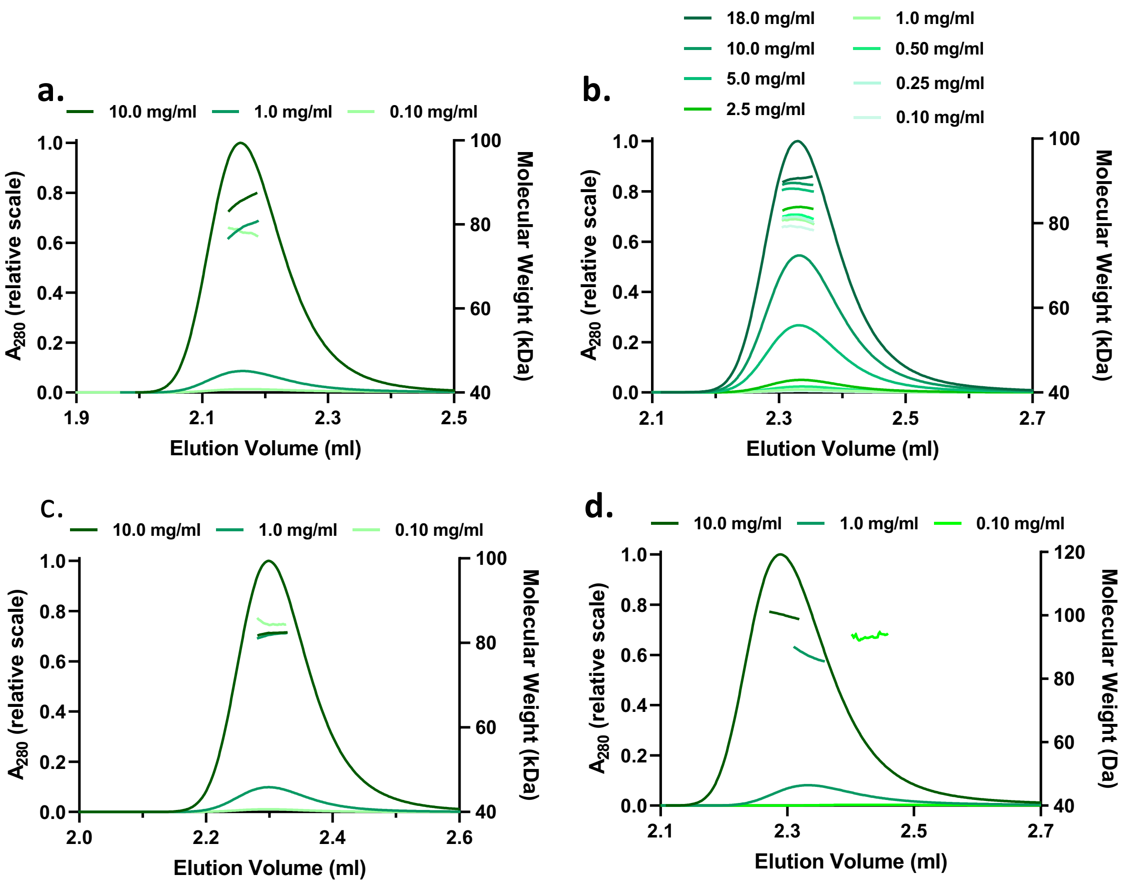

Supplementary Figure S4. SEC-MALS. Results of the SEC-MALS measurements showing the relative elution profiles and corresponding molecular weights of *Vc*ParD at concentrations varying from 18 mg/ml to 0.1 mg/ml. The experiments were performed in following buffer conditions: **a.** 20 mM Tris pH 8.0, 50 mM NaCl, 1mM TCEP; **b.** 20 mM Tris pH 8.0, 150 mM NaCl, 1mM TCEP; **c.** 20 mM Tris pH 8.0, 500 mM NaCl, 1mM TCEP; **d.** 50 mM NaAc pH 5.6, 150 mM NaCl, 1mM TCEP.

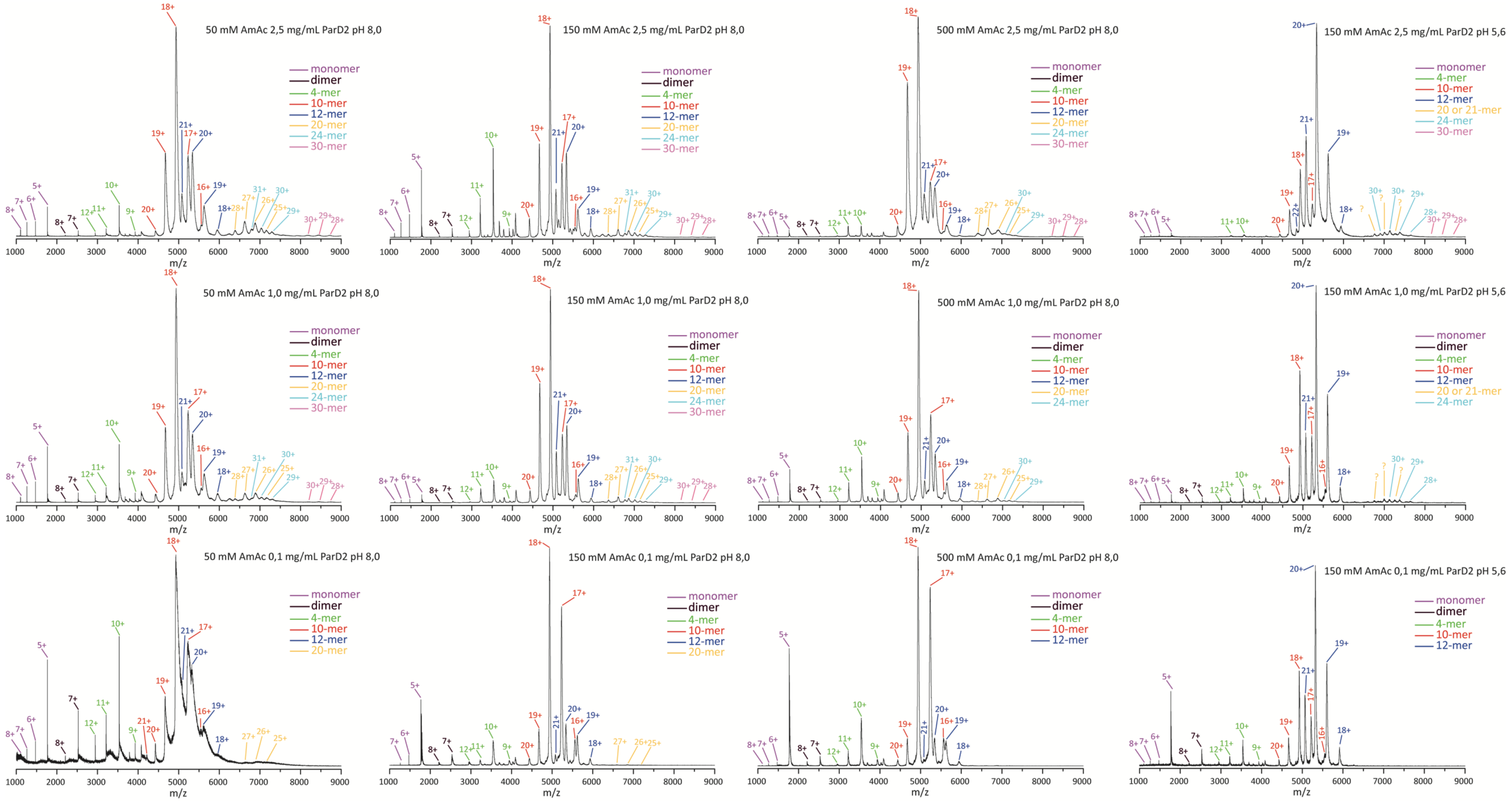

Supplementary Figure S5. Native mass spectrometry. Native MS spectra are shown for VcParD2 at different concetntrations, ionic strengts and pH. In all cases the dominant species are 10-mer and 12-mer althoughtheir relative abundance can vary.

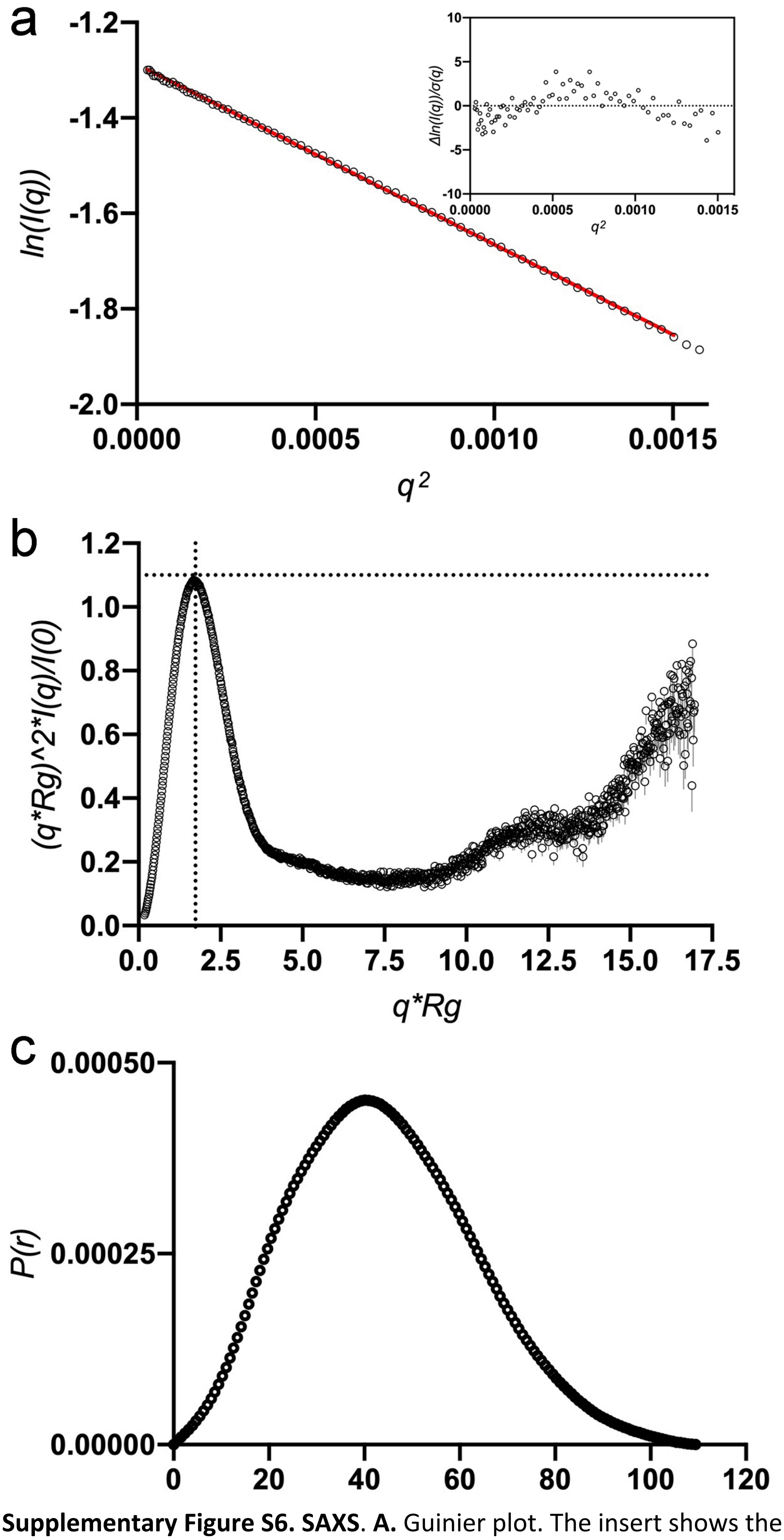

Supplementary Figure S6. SAXS. **A.** Guinier plot. The insert shows the residuals from the linear fit. **b**. Kratky plot. **c.** P(r) function.

Supplementary Table S1.

concentration (mg/ml)	Ionic strength	Buffer	Technique	Molecular weight (kDa)
0.15			SEC	170
12	150 mM NaCl	20 mM Tris pH 8.0, 1 mM TCEP	SAXS (Porod)	109
18	150 mM NaCl	20 mM Tris pH 8.0, 1 mM TCEP	SEC-MALS	91.0 ± 0.7%
10	150 mM NaCl	20 mM Tris pH 8.0, 1 mM TCEP	SEC-MALS	89.1 ± 0.6%
5	150 mM NaCl	20 mM Tris pH 8.0, 1 mM TCEP	SEC-MALS	87.7 ± 0.8%
2.5	150 mM NaCl	20 mM Tris pH 8.0, 1 mM TCEP	SEC-MALS	83.4 ± 0.7%
1	150 mM NaCl	20 mM Tris pH 8.0, 1 mM TCEP	SEC-MALS	79.9 ± 1.2%
0.5	150 mM NaCl	20 mM Tris pH 8.0, 1 mM TCEP	SEC-MALS	80.4 ± 0.7%
0.25	150 mM NaCl	20 mM Tris pH 8.0, 1 mM TCEP	SEC-MALS	80.6 ± 0.7%
0.1	150 mM NaCl	20 mM Tris pH 8.0, 1 mM TCEP	SEC-MALS	78.5 ± 0.6%
10	50 mM NaCl	20 mM Tris pH 8.0, 1 mM TCEP	SEC-MALS	85.0 ± 0.6%
1	50 mM NaCl	20 mM Tris pH 8.0, 1 mM TCEP	SEC-MALS	79.2 ± 0.6%
0.1	50 mM NaCl	20 mM Tris pH 8.0, 1 mM TCEP	SEC-MALS	77.9 ± 1.7%
10	500 mM NaCl	20 mM Tris pH 8.0, 1 mM TCEP	SEC-MALS	81.9 ± 0.2%
1	500 mM NaCl	20 mM Tris pH 8.0, 1 mM TCEP	SEC-MALS	81.6 ± 0.2%
0.1	500 mM NaCl	20 mM Tris pH 8.0, 1 mM TCEP	SEC-MALS	84.8 ± 2.2%
10	150 mM NaCl	20 mM Na Acetate pH 5.6, 1 mM TCEP	SEC-MALS	100.0 ± 0.6%
1	150 mM NaCl	20 mM Na Acetate pH 5.6, 1 mM TCEP	SEC-MALS	87.6 ± 0.6%
0.1	150 mM NaCl	20 mM Na Acetate pH 5.6, 1 mM TCEP	SEC-MALS	94.2 ± 1.9%
